# Supplementary material for: The Economic Value of Vaccination: Why Prevention is Wealth
Source: J Mark Access Health Policy. 2015 Aug 12;3:10.3402/jmahp.v3.29414. doi: 10.3402/jmahp.v3.29414 (PMC4802700; doi:10.3402/jmahp.v3.29414)
Supplement: Related texts

# Sub article: The economic value of vaccination: why prevention is wealth

**DOI:** 10.3402/jmahp.v3.29284

**Published:** 2015-08-12

**Authors:** Vanessa Rémy, Nathalie Largeron, Sibilia Quilici, Stuart Carroll

**Affiliations:** 1 Sanofi Pasteur MSD, Lyon, France; 2 Sanofi Pasteur MSD, Maidenhead, United Kingdom

Theoretical and empirical evidence has demonstrated that health has a major role to play as a driver for economic growth. Improving health outcomes can have a positive impact on economic outcomes and societal well-being, for example, through longer working lives, higher productivity, improved educational outcomes, social inclusion, and reduced healthcare costs (1).

The economic crisis that started in Europe in 2008 has put tremendous pressure on national budgets, leading to arbitrary cuts with important consequences for healthcare systems and the health of European citizens. It has been predicted that the number of people aged ≥65 years will almost double, and those aged ≥80 years will triple, by 2060 in the European Union (EU) (2). The combination of the difficult economic situation and the demographic pressure results in important challenges to meet the higher demand for healthcare and to adapt healthcare systems to the needs of an ageing population while keeping them sustainable.

Key opinion leaders, scientific experts, as well as governments and national budget holders face the same dilemma: how to spend the limited financial resources dedicated to healthcare more efficiently for the benefit of the population. One approach to do this, while improving the health of Europeans, is to concentrate our efforts on keeping people healthy, rather than waiting to treat them once they become ill. Prevention is one of the best means of helping Europeans to live healthier and longer, thereby increasing European productivity.

In Europe, the national budgets allocated to healthcare represent an average of 9.0% of the gross domestic product (GDP), although on average, only 3% of this budget is dedicated to prevention (3). The preventative healthcare budget is allocated to diverse areas such as smoking cessation, reduction of alcoholism, improved nutrition, encouraging physical activity, and higher uptake of vaccinations. This budget has decreased in many EU countries over recent years, except in 2009 due to the pandemic flu crisis (Fig. 1) (3). Generally, preventative programmes are most vulnerable to budget cuts and restrictions since their benefits are not always immediately identifiable. These cuts often have a short-term focus and do not affect directly identifiable patients, but they do, however, affect future public health. Thus, new healthcare models are needed to change the focus of current systems from illness management to healthcare management integrating cost-effective preventative interventions.

**Fig. 1:** Evolution of healthcare prevention budgets in Europe (3).

Although a minor fraction of the healthcare budget is allocated to vaccination programmes (e.g., equivalent to about 0.3% of healthcare expenditure in France), they have a central role in prevention policies (4). The recent European Council conclusions on ‘vaccination as an effective tool in public health’ highlighted that an evidence-based, cost-effective, safe, and efficient vaccination system is an integral part of a well-functioning health system (5). Vaccines are among the most successful and cost-effective public health tools for preventing diseases and death. They have led to the eradication of smallpox, the elimination of polio from most continents, and the control of other diseases, including diphtheria, tetanus, pertussis, rubella, and hepatitis B. The impact of vaccines can be measured not just in terms of public health, but also in economic terms: reducing healthcare costs, decreasing lost labour force productivity, and contributing to social and economic development (6, 7).

In December 2014, The Council of European Ministers of Health updated the Europe 2020 Strategy, to acknowledge the importance of investment in health as a contributor to economic growth, highlighting the role of preventative actions, including vaccination against communicable diseases, in strengthening cost-effectiveness (8). Vaccination and the vaccine industry are undeniably key contributors to the smart, sustainable, and inclusive growth objectives for Europe 2020. With about 80% of the world's vaccine production occurring in Europe, the vaccine industry represents a key contributor to employment and productivity with a higher than average proportion of employees having tertiary-level education. Vaccine-related research and development is also one of the highest (15% of sales) among all industries (9). Lastly, infectious disease control and vaccination coverage may act as an effective safeguard against poverty and health inequalities.

Governments and policy makers will need to acknowledge that prevention through vaccination involves low levels of investment relative to the substantial benefits procured for European citizens and the European economy. Several countries, such as the United States, Canada, and Australia, have begun to consider vaccination as pivotal in their prevention programmes. Taking into account the full economic benefits of vaccination would allow positioning prevention as one of the best ways to identify efficiency gains.

This special issue in the *Journal of Market Access and Health Policy* is made up of a series of seven articles that aim to provide policy makers with robust evidence on the economic benefits of vaccination in Europe, from different angles and perspectives. We acknowledge the limitations of this series in focusing on the benefits of vaccination from an economic perspective and, thus, may not fully address the potential drawbacks of vaccines, in particular from a clinical perspective. The objectives of this special issue are threefold:

- To demonstrate the full economic value of vaccination based on published examples;
- To describe the contribution that vaccination can make to healthcare systems’ sustainability and efficiency and also to the wider economy;
- To launch a call for action for developing and implementing tools that allow the full economic value of vaccination to be taken into consideration.

Together, the authors would like to make a call to each country, each region, and each health constituency to appreciate the level of budget that they allocate to this important public health intervention. Indeed, there is a strong case for renewed European commitment to vaccination that requires action on several fronts:

- ***Shift in mind and in budget***: Given the undeniable importance of vaccination for public health, there is a need to secure an appropriate level of budget to guarantee populations’ access to vaccination;
- ***Shift in communication***: There is a need to communicate clearer and more compelling messages about the value of vaccination to governments, policy makers, as well as healthcare professionals and the lay public;
- ***Shift in vaccines evaluation***: There is a need to consider the global economic benefits from vaccination. Economic evaluations should move beyond assessing only the effect of vaccination on health and medical costs at the individual level, and address the broader economic value to society.

## References

[1] Barnighausen T, Bloom DE, Cafiero-Fonseca ET, O'Brien JC (2014). Valuing vaccination. Proc Natl Acad Sci U S A.

[2] Economic Policy Committee and European Commission (EPC/EC) (2011). The 2012 Ageing Report: Underlying assumptions and projection methodologies. European Economy, No. 4/2011, Directorate General Economic and Financial Affairs, European Commission 2011.

[3] OECD Health at a glance 2013: OECD indicators.

[4] Zaidman C, Roussel R, Le Garrec MA, Bouvet M, Solard J, Mikou M (2014). Comptes Nationaux de la Santé 2013.

[5] Council of the European Union Council conclusions on vaccinations as an effective tool in public health.

[6] Leroy O, Geels M, Korejwo J, Dodet B, Imbault N, Jungbluth S (2014). Roadmap for the establishment of a European vaccine R&D infrastructure. Vaccine.

[7] European Network of Vaccine Research and Development (TRANSVAC) Forwards to a European vaccine R&D infrastructure. A roadmap for success.

[8] Council of the European Union Investing in health: The ‘missing dimension’ of the Europe 2020 Strategy.

[9] Vaccines Europe Vaccines’ contribution to Europe's future.

# Sub article: Vaccination: the cornerstone of an efficient healthcare system

**DOI:** 10.3402/jmahp.v3.27041

**Published:** 2015-08-12

**Authors:** Vanessa Rémy, York Zöllner, Ulrike Heckmann

**Affiliations:** 1 Sanofi Pasteur MSD, Lyon, France; 2 Hamburg University of Applied Sciences, Hamburg, Germany; 3 Sanofi Pasteur MSD, Berlin, Germany

**Keywords:** vaccination, economic analysis, cost-effectiveness, cost-saving, public health

## Abstract

Vaccination has made an important contribution to the decreased incidence of numerous infectious diseases and associated mortality. In 2013, it was estimated that 103 million cases of childhood diseases in the United States had been prevented by the use of vaccines since 1924. These health effects translate into positive economic results, as vaccination can provide significant savings by avoiding the direct and indirect costs associated with treating the disease and possible long-term disability. A recent US study estimated that every dollar spent on childhood vaccination could save US$3 from a payer perspective and US$10 from a societal perspective. The first vaccines set a high standard from a public health ‘return on investment’ perspective, because they are highly cost-saving. Today, however, where only a few healthcare interventions are considered to be cost-saving, the challenge that decision-makers typically face is to identify such healthcare interventions that are deemed cost-effective, that is, provide extra benefit at a reasonable extra cost. Some of the newer vaccines provide a solution to some of today's important health issues, such as cervical cancers with human papillomavirus vaccines, or debilitating diseases with herpes zoster vaccines. These recent, more expensive vaccines have been shown to be cost-effective in several economic analyses. Overall, vaccination can still be regarded as one of the most cost-effective healthcare interventions.

During the 20th century, improved sanitation, nutrition, and the widespread use of antibiotics as well as vaccines have all contributed to the decreased incidence of numerous diseases and associated mortality. Vaccination was one of the public health measures that had the greatest impact on the reduction of the burden from infectious diseases and associated mortality, especially in children. It is estimated that, each year worldwide, vaccines prevent up to 3 million deaths (1, 2). In 1980, vaccination was responsible for the global eradication of smallpox for the first time in history. Vaccination has also led to the elimination of wild-type poliovirus in the Americas in 1990, in the Western Pacific Region in 2000, and in the European Region in 2002, and to the elimination of *Haemophilus influenza* type B (Hib) within a few years of introduction of conjugate Hib vaccines in many countries. Currently, there are more than 40 vaccines available for the prevention of 25 vaccine-preventable diseases (3). These health effects translate into positive economic results, and vaccination is commonly recognised as one of the most cost-effective public health investments (4, 5). However, most vaccines are considered to be underused; furthermore, they are probably undervalued (4). This article aims at examining the public health and economic impact of vaccination in industrialised countries, with a specific focus on Europe.

## Contribution from vaccination to public health

Vaccination has made a fundamental contribution to the prevention of numerous infectious diseases. Worldwide, it is estimated that vaccines prevent, annually, 5 million deaths caused by smallpox, 2.7 million cases of measles, 2 million cases of neonatal tetanus, 1 million cases of pertussis, 600,000 cases of paralytic poliomyelitis, and 300,000 cases of diphtheria (6).

In industrialised countries, several infectious diseases have been controlled and, in some cases, eliminated through routine vaccination. The generally high level of vaccination coverage has led to a dramatic decline in the reported incidence of many vaccine-preventable infectious diseases (Fig. 1) (7). A comparison between the period prior to the implementation of national vaccination recommendations in the United States and 2006 showed a greater than 99% decline in the number of cases of diphtheria (100%), measles (99.9%), paralytic poliomyelitis (100%), and rubella (99.9%). A greater than 92% decline in cases and a 99% or greater decline in deaths were shown for mumps, pertussis, and tetanus (8, 9). In 2013, it was estimated that 103 million cases of childhood disease in the United States had been prevented by the use of vaccines since 1924, of which 26 million cases in the past decade alone (10). A similar trend has been observed in Europe (Table 1) (9, 11). In France, diphtheria, tetanus and polio, BCG (tuberculosis), and pertussis vaccines were estimated to be responsible for saving more than 400,000 years of life (4).

**Fig. 1:** Comparison of the estimated annual morbidity in the United States in the pre- and post-vaccine eras (7).

**Table 1:** Number of reported cases of vaccine-preventable diseases in the European region based on data from the WHO vaccine-preventable disease monitoring system

	1980	2000	2011	2012	2013
Diphtheria	608	1,585	33	32	32
Measles	851,849	37,421	37,073	26,982	25,375
Mumps	No data	243,344	27,448	38,141	35,075
Pertussis	90,546	53,675	34,432	56,941	27,824
Polio	549	0	0	0	0
Rubella	No data	621,039	9,672	30,509	39,614
Rubella (CRS)	No data	48	7	60	50
Tetanus	1,715	412	197	194	93

From Refs (9, 11). CRS: congenital rubella syndrome; Full database available in Ref. (11).

Another example is *H. influenzae* type b (Hib) invasive disease, which was the leading cause of childhood meningitis and was associated with high death rates and sequelae. Before a Hib vaccine was available, an estimated 445,000 cases of invasive Hib disease occurred in children under 5 years of age globally, each year, 115,000 of which resulted in death. The incidence of Hib meningitis in Europe has been reduced by more than 90% in less than 10 years because of vaccination (4).

## Vaccination represents a valuable investment in health with positive economic return

Whether the benefits are reported in terms of avoided deaths, life-years saved, disability-adjusted life years (DALYs) avoided or quality adjusted life years (QALYs) gained, vaccination is universally considered to provide important public health benefits (12). These health effects translate into positive economic outcomes. Vaccination can provide significant savings by avoiding the health costs associated with treating diseases. Table 2 summarises the results of a US study estimating the direct and indirect (i.e., loss of productivity) costs savings for several vaccine-preventable diseases (13).

**Table 2:** Direct and indirect savings from vaccination

Disease	Comparative savings	Direct or indirect savings (US$)
Smallpox^a^	NA	300 million in direct costs per year
Polio^b^	NA	13.6 billion in total savings world wide by 2040 700 million in the United States between 1991 and 2000
Measles	Treating one child with measles costs 23 times the cost of vaccinating one child against measles	10 per disability-adjusted life-year (DALY)
Cholera	NA	770 million lost in seafood exports in Peru, 1991
Malaria	NA	100 billion GDP lost annually in sub-Saharan Africa
MMR	For every US$ spent on MMR vaccine, more than US$21 is saved in direct medical care costs	100 million in direct medical costs from 1989 to 1991 for measles outbreaks
DTaP	For every US$ spent on DTaP vaccine, US$24 is saved	23.6 billion in direct and indirect costs without DTaP vaccines
Hib	For every US$ spent on Hib vaccine, more than US$2 is saved	5 billion in direct costs and 12 billion in indirect costs incurred in the United States

From Ref. (13). NA: not available; MMR: measles–mumps–rubella; DTaP: diphtheria–tetanus–acellular pertussis; Hib: *H. influenza*e type b.

a Based on eradication of smallpox in 1977;

b based on eradication of polio by 2005; calculation details available in Ref. (13).

The economic impact of vaccination programmes has been evaluated through different economic indicators, such as benefit–cost ratio (BCR=total discounted benefits divided by total discounted programme costs, if >1: benefits outweigh the costs), the net benefit (total discounted benefits minus total discounted costs) and return on investment (ROI=net benefit divided by costs, if >0: benefits exceed the costs), as illustrated in the following examples. Current childhood vaccines against diphtheria, tetanus, pertussis, Hib, polio, measles, mumps, rubella, and hepatitis B, when considered together, were estimated to have a BCR of more than 5:1 for direct costs and 17:1 for societal costs (14). A recent US study confirmed the pattern of this finding, estimating that every dollar spent on childhood vaccination saves US$3 from a payer perspective (i.e., direct costs) and US$10 from a societal perspective (i.e., direct and indirect costs; Table 3) (15). In the United States, the diphtheria, tetanus, and pertussis (DTP) vaccine has resulted in direct and indirect cost savings of US$23.6 billion, with a corresponding BCR of 27:1 (16). In another US study, it was estimated that the net benefit for 60 years of investment in polio vaccine was six times higher (approximately US$180 billion) than the total investment over the same period (approximately US$36.4 billion) (17). A European review, taking the UK as an example, demonstrated that for every euro spent on targeted influenza vaccination for the elderly, €1.35 savings were generated in terms of reduced medical spending elsewhere (18) in the healthcare system. In Europe, an Italian study reported that universal hepatitis B childhood vaccination would have a positive economic impact 20 years after its implementation (19). The ROI was estimated to be almost 1 from the National Health Service perspective, and the BCR slightly less than 1 from the societal perspective, considering only the first 20 years after the start of the programme. With a longer term horizon, both the ROI and BCR values were estimated to be positive (2.78 and 2.47, respectively). The hepatitis B vaccination programme in Italy is a clear example of the massive impact that universal vaccination can have on the medium-to-long-term, when healthcare authorities are wise enough to invest in prevention (19).

**Table 3:** Summary of an economic evaluation of the routine childhood vaccination programme in the US in 2009

Childhood vaccination programme	Payer perspective	Societal perspective
Costs saved	20.3	76.4
Costs of routine immunization programme	6.7	7.5
Net cost savings	13.5	68.8
Benefit–cost ratio	3.0	10.2

Costs are given as 2009 billion US$. *Note*: Calculations based on population-based vaccination coverage, published vaccine efficacies, historical data on disease incidence before vaccination, and disease incidence reported during 2005–2009. Programme costs included vaccine, administration, vaccine-associated adverse events, and parent travel and work time lost. Three percent annual discount rate (15).

Investments in infectious disease eradication have also proven highly valuable. For example, the World Health Organisation invested more than US$300 million over 11 years in the Intensified Smallpox Eradication Programme (1967–1979). This investment has paid back many times by saving human lives and by the elimination of downstream costs for vaccines, treatment, and international surveillance activities. The annual savings from smallpox eradication are estimated to be more than US$2 billion each year; these savings have been used for other pressing health issues (4). A similar trend could be expected if polio eradication were achieved: ‘The world as a whole is expected to save US$1.5 billion a year once vaccination is discontinued, of which the United States would save about US$230 million’ (20).

## Modern vaccines: continued good value for money

Vaccination is often considered as the most cost-effective public health intervention after clean water (4, 21). The first vaccines set a high standard because they were cost-saving, i.e., the money invested in vaccination programmes was completely offset by the treatment costs avoided. These vaccines were introduced in an environment of poorer quality of population health and sanitary conditions, with a very high incidence and morbidity of infectious diseases. Today's new vaccines are available in a better health environment and represent a solution to our health issues today, such as cancers or debilitating diseases. Compared with the original vaccines, these new vaccines are more costly, partly as a result of their more advanced and complex, patent-protected technologies, such as recombination techniques, carrier proteins, and adjuvants (22). In addition, recent analyses suggest that increased regulatory oversight is another factor driving up the price of new vaccines (22, 23). However, economic analyses have reported that, despite their higher costs, new vaccines have been found to be cost-effective (according to commonly used thresholds in Europe ranging from €20,000 to €50,000/QALY), meaning that they provide good health value at a cost deemed reasonable, according to payers’ willingness to pay (24). For example, a systematic review analysed 15 published economic evaluations on the human papillomavirus (HPV) vaccine performed in Europe, of which 10 were industry-sponsored, while 5 were not (25). Interestingly, the authors reported that nine sponsored studies as well as the five non-sponsored studies were favourable to HPV vaccination cost-effectiveness, while one of the 10 industry-sponsored studies was not (25). In another systematic review of the cost-effectiveness of zoster vaccination, all but one of the studies included in the review concluded that most vaccination scenarios were cost-effective (26). However, comparisons between cost-effectiveness studies may be difficult because of variability and uncertainty around model assumptions (i.e., perspective, model design, time horizon, comparators, etc.) or input data applied between studies and countries. For example, a systematic review reported that rotavirus vaccination was found to be cost-effective in developing countries but that conclusions varied between studies in developed countries (27). Rotavirus vaccination was likely to be cost-effective under some scenarios, such as inclusion of herd protection and adoption of a societal perspective, demonstrating the need to thoroughly evaluate studies’ comparability before drawing any conclusion.

## Conclusion

Vaccination has made a fundamental contribution to the decreased incidence of numerous infectious diseases and associated mortality. These health effects translate into positive economic outcomes for healthcare systems and to society as a whole. Vaccines are generally regarded as one of the most cost-effective public health measures available. However, they are often undervalued and/or underused, though for different reasons: undervalued, paradoxically, in some parts of the world where increased vaccination coverage could provide significant benefit; underused, in other parts of the world where the high standards of health and healthcare seem to be have led to the achieved vaccine-borne benefits being taken for granted, in these societies at risk of complacency.

The under-utilisation of vaccines in industrialised countries could be seen as vaccination being a victim of its own success, leading people to underestimate the seriousness of vaccine-preventable diseases and the benefits of vaccination, and, instead, to have concerns regarding the side effects of vaccines. As for any biological or medicinal product, adverse reactions due to vaccines, although extremely rare, exist. For example, the risk a child will have a severe reaction after receiving the MMR (Measles, Mumps, and Rubella) or DTaP vaccine is less than 1 in 1,000,000 (28). Additionally, even if vaccination is one of the most cited examples of positive externalities, through herd immunity and reduced transmission of the disease, it may also have potentially negative epidemiological effects such as serotype replacement or shift of disease to older populations. These potential effects should be closely monitored and weighed against the benefits of protecting from severe vaccine-preventable diseases to conclude on the benefit/risk profile of a particular vaccination programme.

In parallel, the ability to reduce the global disease burden with vaccines continues to grow, as new vaccines are developed to prevent other diseases and policy-makers must decide where and how scarce resources are best allocated. Product- and programme-specific attributes such as safety, efficacy, feasibility, and cost-effectiveness play an important role in the basic health system objectives of efficiency, equity, and sustainability. The earlier vaccines set a high standard because they were not only cost-effective, but often even cost-saving, turning decision-making into a relatively straightforward task (requiring only strictly rational behaviour). However, it seems too narrow today to expect that vaccines should be cost-saving (i.e., ‘pay for themselves’), especially in the short-term, since this would suggest that investing in preventive measures is not worthwhile. The new generation of vaccines, despite not always being cost-saving, has been shown to be cost-effective by many research teams, in a multitude of scenarios.

Ultimately, it is the global society and future generations that benefit when all countries make the effort to protect their populations from vaccine-preventable diseases. As such, vaccination programmes need adequate support and recognition of their value for an efficient and timely implementation and realisation of their full potential (23).

## References

[1] (2011). Global alliance for vaccines and immunization. Fact Sheet No. 169.

[2] Ehreth J (2005). The economics of vaccination from a global perspective: Present and future. 2–3 December, 2004, Vaccines: all things considered, San Francisco, CA, USA. Expert Rev Vaccines.

[3] Garcia-Altes A (2013). Systematic review of economic evaluation studies: Are vaccination programs efficient in Spain?. Vaccine.

[4] Ehreth J (2003). The global value of vaccination. Vaccine.

[5] OECD Health at a glance 2013: OECD indicators 2011.

[6] De Melker HE (1999). Seroepidemiology of diphtheria, tetanus, poliomyelitis and pertussis – Evaluation of the National Immunisation Programme in the Netherlands.

[7] Farrant L Vaccine infographic 2012.

[8] Roush SW, Murphy TV (2007). Historical comparisons of morbidity and mortality for vaccine-preventable diseases in the United States. JAMA.

[9] Wicker S, Maltezou HC (2014). Vaccine-preventable diseases in Europe: Where do we stand?. Expert Rev Vaccines.

[10] van Panhuis WG, Grefenstette J, Jung SY, Chok NS, Cross A, Eng H (2013). Contagious diseases in the United States from 1888 to the present. N Engl J Med.

[11] World Health Organisation (2014). WHO vaccine-preventable disease monitoring system, 2014 global summary.

[12] World Health Organisation The global burden of disease: 2004 update 2004.

[13] Ehreth J (2003). The value of vaccination: A global perspective. Vaccine.

[14] Lieu TA, McGuire TG, Hinman AR (2005). Overcoming economic barriers to the optimal use of vaccines. Health Aff (Millwood).

[15] Zhou F, Shefer A, Wenger J, Messonnier M, Wang LY, Lopez A (2014). Economic evaluation of the routine childhood immunization program in the United States, 2009. Pediatrics.

[16] Ekwueme DU, Strebel PM, Hadler SC, Meltzer MI, Allen JW, Livengood JR (2000). Economic evaluation of use of diphtheria, tetanus, and acellular pertuss is vaccine or diphtheria, tetanus, and whole-cell pertussis vaccine in the United States, 1997. Arch Pediatr Adolesc Med.

[17] Thompson KM, Tebbens RJ (2006). Retrospective cost-effectiveness analyses for polio vaccination in the United States. Risk Anal.

[18] Scuffham PA, West PA (2002). Economic evaluation of strategies for the control and management of influenza in Europe. Vaccine.

[19] Boccalini S, Taddei C, Ceccherini V, Bechini A, Levi M, Bartolozzi D (2013). Economic analysis of the first 20 years of universal hepatitis B vaccination program in Italy: An a posteriori evaluation and forecast of future benefits. Hum Vaccin Immunother.

[20] Barrett S (2004). Eradication versus control: The economics of global infectious disease policies. Bull World Health Organ.

[21] OECD Health at a glance 2011: OECD indicators 2011.

[22] Barnighausen T, Bloom DE, Cafiero ET, O'Brien JC (2012). Economic evaluation of vaccination: Capturing the full benefits, with an application to human papillomavirus. Clin Microbiol Infect.

[23] Szucs T, Quilici S, Panfilo M (2015). From population to public institutions: What needs to be changed to benefit from the full value of vaccination. J Market Access Health Policy.

[24] Drummond M (2007). NICE: A nightmare worth having?. Health Econ Policy Law.

[25] Koleva D, De Compadri P, Padula A, Garattini L (2011). Economic evaluation of human papilloma virus vaccination in the European Union: A critical review. Intern Emerg Med.

[26] Szucs TD, Pfeil AM (2013). A systematic review of the cost effectiveness of herpes zoster vaccination. Pharmacoeconomics.

[27] Aballea S, Millier A, Quilici S, Caroll S, Petrou S, Toumi M (2013). A critical literature review of health economic evaluations of rotavirus vaccination. Hum Vaccin Immunother.

[28] CDC Vaccines & immunizations: possible side-effects from vaccines 2015.

# Sub article: Role of vaccination in the sustainability of healthcare systems

**DOI:** 10.3402/jmahp.v3.27043

**Published:** 2015-08-12

**Authors:** Nathalie Largeron, Pierre Lévy, Jürgen Wasem, Xavier Bresse

**Affiliations:** 1 Sanofi Pasteur MSD, Lyon, France; 2 LEDa-LEGOS, Université Paris-Dauphine, Paris, France; 3 Institute of Health Care Management and Research, Universität Duisburg, Essen, Germany

**Keywords:** vaccination, healthcare resource use, cancers, costs, nosocomial infections

## Abstract

The use of vaccines to prevent diseases in children, adults, and the elderly results in fewer medical visits, diagnostic tests, treatments, and hospitalisations, which leads to substantial savings in healthcare costs each year in Europe and elsewhere. Vaccines also contribute to reducing resource utilisation by preventing nosocomial infections, such as rotavirus gastroenteritis, which can increase hospital stays by 4–12 days. Vaccination also has an important role in the prevention of cancers with, for example, human papillomavirus or hepatitis B vaccines. Since the financial impact of cancer is high for patients, healthcare systems, and society as a whole, any cases prevented will reduce this impact. Newer vaccines, such as the herpes zoster vaccine, can provide an answer to unmet medical needs by preventing and reducing the severity of shingles and associated post-herpetic neuralgia, which are difficult conditions to treat. Thus, in the context of increasing pressure on healthcare budgets, vaccination can contribute to the sustainability of healthcare systems through reduced and more efficient use of healthcare resources.

In the past decades, vaccination has dramatically reduced the incidence of several infectious diseases that were responsible for much suffering and deaths. The impact of vaccination has been demonstrated by the estimate that during the ‘vaccines for children era’ (1994–2013), the total number prevented by routine childhood vaccinations in the USA was more than 322 million cases of infectious diseases, 21 million hospitalisations, and 731,700 deaths (1). Thus, vaccination has made a substantial contribution to the sustainability of healthcare systems by reducing the burden of frequent infectious diseases and associated resource use. There is an increasing number of vaccines protecting against a range of infections affecting not just children but also adults and the elderly. In this article, we will examine how an effective use of these vaccines can further reduce the burden on healthcare systems by reducing healthcare resource utilisation, preventing severe diseases such as cancers and nosocomial infections, and answering unmet medical needs.

## Reduction of hospitalisations, ambulatory care visits, and medical interventions

### In children

Today, children in Europe routinely receive vaccines that protect them from more than a dozen diseases. As presented in the first article of this special issue, childhood vaccination resulted in a substantial decrease in the incidence of numerous infectious diseases and associated mortality such as diphtheria, tetanus and polio, tuberculosis, pertussis, measles, mumps, and rubella (2). For example, prior to introduction of the conjugate vaccine, Haemophilus influenzae b (Hib) was the leading cause of childhood meningitis, pneumonia, and epiglottitis, causing an estimated 20,000 cases per year in the early 1980s in the United States, mostly in children under 5 years old. Since routine vaccination began between 1980 and 1990, the incidence of Hib diseases has declined by greater than 99%. Similar reductions in disease occurred after introduction of the vaccine in Western Europe and developing countries. These ‘traditional’ vaccines have become the cornerstone of efficient healthcare systems throughout the world and have been shown to be highly cost-saving (2). Since the late 1990s, new vaccines, such as rotavirus (RV), meningococcal, pneumococcal conjugate, or varicella vaccines have become available. Although access to these new vaccines is not homogeneous across Europe, they have been shown to reduce the costs associated with hospitalisations and outpatient visits (Table 1) (3–13).

**Table 1:** Estimated human and economic burden in Europe of some new preventable diseases

	Annual burden before vaccine introduction	Cost per case
Rotavirus gastroenteritis	In Europe in children <5 years (3) ‐ 3.6 million episodes ‐ 87,000 hospital admissions ‐ 700,000 GP consultations ‐ 231 deaths	Societal perspective (including direct medical, direct non-medical, and indirect costs) in 2004–2005 in Belgium, France, Germany, Italy, Spain, Sweden, and UK (5) ‐ From €166 to €473 in the primary care setting ‐ From €334 to €770 in the emergency department setting ‐ From €1,525 to €2,101 in the hospital setting ‐ Mean number of work days lost by parents: 2.3–7.5 days
Meningitis C	In the UK: 955 cases in 1998 (7)	In the UK: £8,413 per case (8)
Invasive pneumococcal diseases (meningitis and pneumonia)	In the UK: ‐ More than 5,000 cases of IPD are diagnosed each year in England in all age groups (12) ‐ From July 2005 to June 2006, 797 cases of invasive pneumococcal disease in children <5 years (10)	In the UK: Meningitis: 2,274 £ per case (11) Post-meningitis sequelae: £3,335/year
External genital warts	In Europe: 600,000 new cases (14)	In France: €483 per case (payer perspective)
HPV-related cancers	In Europe: (46) ‐ Between 267,000 and 510,000 cervical precancerous lesions ‐ 35,000 cervical cancers ‐ 6,400 anal cancers ‐ 3,400 vulvar and vaginal cancers ‐ 1,300 penile cancers ‐ 11,000 head and neck cancers	In the UK: From £12,700 (penile cancer) to £16,400 (cervical cancer) per case (payer perspective) (13)
Zoster	In Europe: 1.7 new cases (54)	In Germany: €388 to €729 per case (payer perspective) (54)

For example, in November 1999, the United Kingdom was the first EU country to introduce mass vaccination against group C meningococcal disease (8). Invasive meningococcal disease is a serious bacterial infection mainly affecting young children. The disease progresses rapidly, has a fatality rate of 5–10%, and a considerable proportion of survivors have long-term disabling sequelae such as deafness, neurological impairments, and amputation. Since 2000, this vaccination programme has prevented over 9,000 cases of serious disease and more than 1,000 deaths (7). This represents considerable savings since the cost of treating these diseases was estimated at nearly £10 million, before vaccine introduction (8).

RV gastroenteritis (RVGE) is currently the most common cause of severe gastroenteritis in infants and young children in both developed and developing countries, leading to more than 87,000 hospitalisations per year in Europe (Table 1) (3). The direct yearly costs are estimated at almost €63 million in France and €67–80 million in Italy (5, 6). Two RV vaccines obtained market authorisation in Europe in 2008 and 2009. Belgium, Luxembourg, Austria, Finland, the UK, and more recently Germany have implemented universal RV vaccination. In the UK, the programme was launched in summer 2013 for children below 1-year-olds. The first full year's data on the impact of this national infant RV immunisation programme confirmed that the programme has been successful, with a 71% reduction in the number of cases. Significant reductions were also observed in numbers of General Practice (GP)-reported cases and in those reported by emergency departments (16).

Episodes of invasive pneumococcal pneumonia (IPD) in children make substantial demands on hospital healthcare and financial resources. For example, it was estimated that a child hospitalised for IPD in Spain had a median length of stay of 11.0 days, with an associated cost of €4,533 per stay. A substantial part of these costs can be avoided with a universal pneumococcal (PCV13) childhood vaccination programme and early management of complications (14).

A routine varicella vaccination programme could also have an important economic impact. It has been estimated that 68% of hospitalisations and 57% of deaths could be prevented with a 90% vaccination uptake in Italy (17). Vaccination costs would be more than offset by the reduction in varicella treatment costs within the first years after implementation. Despite this positive economical impact, varicella vaccine recommendations in Europe are heterogeneous, with only five countries where varicella vaccination is universally recommended for children at national level and two countries at regional level (18).

Influenza is a highly contagious disease that is responsible for 3–5 million cases of severe illness each year globally, with a high attack rate observed among children (9). A comprehensive systematic review, including 50 publications on the influenza burden in children in Europe, estimated that up to 20% of children aged 0–11 months with influenza are hospitalised with a mean length of stay of between 1.8 and 7.9 days (9). Therefore, successful implementation of these childhood vaccination programmes could lead to a substantial decrease in healthcare resource use, which should be balanced against the programme cost in the assessment of the programme efficiency.

### In adolescents and young adults

External genital warts (EGWs) are a sexually transmitted infection caused by various strains of human papillomavirus (HPV) that tend to infect particularly teenagers and young adults early after their sexual debut. It has been estimated that, in Europe, almost 600,000 new cases of EGWs occur each year (19).

The economic and human burden of EGWs is high (20). The average treatment cost and total annual direct cost of genital warts were estimated at €483 and €23 million, respectively, in France (21). The quadrivalent HPV vaccine protects against HPV types 16 and 18, as well as 6 and 11; these two latter types represent the causal pathogen for 90% of genital warts. A vaccination programme is, therefore, expected to have a significant impact on the occurrence of genital warts and their associated treatment cost. This assumption has been confirmed in countries such as Australia, which have implemented national vaccination programmes (22, 23).

A significant reduction in pertussis morbidity and mortality in infants and children has been observed where preschool and primary school pertussis vaccination has been implemented (24). However, the disease persists in infants who are too young to be vaccinated, and among adolescents and adults who have lost their disease- or vaccine-induced immunity against the disease or who were not vaccinated during childhood. The re-emergence of pertussis could be limited by a booster vaccination for adolescents and adults. Many countries (e.g., France, Germany, the USA, and Canada) have already integrated a pertussis booster for adolescents and/or adults into their current schedule. The results from a literature review showed that economical models converge towards the same conclusion, that is, that the pertussis booster vaccination is economically valuable when used in an appropriate context (24).

### In adults and elderly

Adult vaccination programmes have been slower to evolve in contrast to childhood vaccination programmes. It is necessary to fully appreciate the value of preventing these diseases to improve the uptake of adult vaccination.

A recent modelling study provided an illustration of the annual public health and economic benefits of influenza vaccination in Europe (25). Today, seasonal flu vaccination already imparts substantial annual health and economic benefits and prevents each year an average of 1.6–2.1 million cases, 45,000–66,000 hospitalisations, 25,000–37,000 influenza-related deaths, and €153–€219 million in healthcare costs (GP visits & hospitalization).

Seasonal influenza vaccination is recommended for approximately 180 million Europeans considered at risk (based on the WHO definition); however, only 44% of them (80 millions) actually receive an annual influenza immunisation, far from the 75% target coverage rate set by the EU Council Recommendation in 2009 (26). With the development of new and improved vaccines, additional benefits will be achieved in the future but today, full implementation of the 75% target coverage rate in the recommended target groups could immediately reduce the burden of seasonal influenza infections by an additional 1.6–1.7 million cases, 24,000–31,000 hospitalisations, 10,000–14,000 influenza-related deaths, and €77–€99 million of influenza-related additional healthcare costs saved yearly (25).

Until recently, influenza vaccines contained three influenza strains, two A strains, and one B strain. These strains are adapted every year to correspond to the circulating strains identified through the World Health Organisation's (WHO) surveillance system (27). There are two A strains, because two co-circulation of A strains have been observed in a given influenza season unlike, until recently, B strains for which only one strain was observed (28). However, in recent seasons the co-circulation of B strains has been increasingly observed and this has led to the WHO issuing recommendations to include four strains in seasonal influenza vaccines, that is, two A strains and two B strains (27). These new quadrivalent influenza vaccines (QIV) that are now commercially available can also play a role in the reduction of healthcare costs. An analysis conducted to quantify the potential public health impact of a QIV vaccine strategy compared with the current trivalent vaccine (TIV) strategy in the United States estimated that over 10 years (from 1999 to 2009), QIV could have prevented up to 2,741,575 cases of influenza, 21,440 hospitalisations, and 1,371 influenza-related deaths in the United States (29). Based on these estimates, it was estimated that over these 10 influenza seasons, QIV could have resulted in substantial cost savings of $3.1 billion to society and $292 million to third party payers if QIV had been used instead of TIV, at the same cost (30). Additionally, the burden and associated healthcare and societal costs from other vaccine-preventable diseases such as pneumococcal, herpes zoster, and pertussis are also substantial.

It was recently estimated that the economic burden from four major adult, vaccine-preventable diseases (influenza, pneumococcal, herpes zoster, and pertussis) in those aged ≥50 years was more than 26.5 billion US$ (medical and indirect costs) in the United States in 2013 (31). Herpes zoster, influenza, and pneumococcal diseases were responsible for 19, 60, and 19% of these costs, respectively (Fig. 1) and medical costs accounted for 80, 91, 37, and 42% of total influenza, pneumococcal, zoster, and pertussis costs, respectively (31). Broadening adult vaccination programmes beyond influenza may, therefore, help reduce the overall burden of disease.

**Fig. 1:** Incidence and total costs of four major adults vaccine-preventable diseases in the United States (2013).

## Reduction in treatment prescription

Preventing diseases through vaccination also leads to a reduction in the consumption of medications. For example, medication use is high in children who have influenza; in addition to antipyretics and other symptomatic treatments, antibiotics were prescribed to 43% of children in primary care-based studies (9).

Vaccines that can prevent diseases, such as influenza, pneumococcal diseases, and shingles, in the elderly are likely to reduce not only the costs associated with medication consumption but also the costs associated with their side-effects. For example, a Dutch study of 84 patients with postherpetic neuralgia (PHN – a well-known complication of shingles) reported that 89% of patients took prescription medications such as antidepressants, opioids, various analgesics as well as antiepileptic medicines (32). The elderly are highly susceptible to side-effects from medications, partly because they generally take more than one medication, and there is often no dose adjustment (33). Polypharmacy is known to be associated with negative health outcomes and a major cause of drug interactions and safety problems in this age group (34).

## Prevention of nosocomial infections

Vaccination can also play a role in preventing nosocomial infections. For example, RV is one of the major aetiological agents for paediatric nosocomial diarrhoea, responsible for between 31 and 87% of cases (35). Nosocomial RVGE cases prolong hospital stays by 4–12 days and can lead to substantial additional costs (36). In Italy, costs resulting from nosocomial RV infection have been estimated at more than €8 million per year (37). Therefore, prevention of RV infection by vaccination could have a positive impact not only by reducing the number of children hospitalised for gastroenteritis, but also by reducing the number of nosocomial infections. The RV burden is significant and includes temporary reduction in the quality of children's lives, increased costs associated with the additional consumption of medical resources (increased length of hospital stay), and constraints on parents’/hospital staff's professional lives (38). A recent UK study reported that the likelihood of nosocomial infection with RV playing a significant role in children's hospital readmissions is high. This has important implications for hospital resources when considering costs and length of hospital stays (38).

*Clostridium difficile* infection (CDI) is also a major cause of nosocomial disease in western countries. CDI results in a significant burden not only for patients, through increased morbidity and mortality, but also for healthcare systems and society in general. On the basis of current incidence rates, the annual cost for the management of CDI is about $800 million in the United States and €3,000 million in Europe (39, 40). Pre-licensure clinical evaluation of the efficacy of two *C. difficile* vaccines is currently on-going. If the vaccines are found to be efficacious, this will provide hope for the future control of this costly bacterial infection.

Vaccines can also reduce the risk of secondary infections, which is relevant not just for those being vaccinated but the wider population. For example, influenza vaccination of healthcare workers (HCWs) has been shown to be associated with a substantial decrease in mortality for elderly patients (41). The cost of not vaccinating frontline HCWs is also significant in terms of missed benefits. It has been estimated that vaccinating each HCW in England and Wales against influenza would result in savings of £12 per vaccination (42).

## Prevention of cancer

In 2012, 2.7 million people were diagnosed with cancer in the European Union (EU27) (43). Nearly, one-fifth of all cancers in the world are caused by infectious agents, including viruses and bacteria. Among the most important infections associated with cancers are: HPVs that can cause most cervical and anal cancers as well as a fraction of oral cancers; hepatitis B virus (HBV) and hepatitis C virus (HCV) that can cause liver cancer; and *Helicobacter pylori* that is a bacterium that can cause stomach cancer (44).

Vaccines are the most effective way of preventing some of these infections. Effective vaccines against HBV have been available for several decades, and more than 90% of countries include HBV vaccination in their childhood immunisation programmes, which has been shown to be responsible for a dramatic reduction in liver cancer (45).

HPV vaccination is also highly effective in preventing infection with the HPV types that cause the majority of cervical cancers and other anogenital cancers in both women and men.

In Europe, between 67,000 and 80,000 new cancers attributable to HPV occur annually in women and men. These include penile and oropharyngeal cancers, for which HPV vaccines are not currently indicated (46, 15). Cervical cancer is the fourth most common cancer in women, diagnosed in approximately 34,000 women annually, and killing about 13,000 each year, in Europe (46). These cancers are responsible for a substantial economic burden in Europe with, for example, an estimated cost to the payers of €240 million in France in 2007 (Fig. 2) (47, 48). Additionally, a recent UK study reported that cervical cancer was not only a cost to the payer (47% of total costs) but also to the state (20% coming from annual loss of income tax and contributions) and to the women (31%) (49, 50).

**Fig. 2:** Estimated costs of HPV-associated cancers in France, payer perspective.

Two HPV vaccines have been commercialised in Europe since 2006. It was estimated that vaccination of 12-year-old girls (70% uptake rate) would lead to a 86% reduction in HPV 16/18-related cervical cancers in females compared with screening alone (51). In addition to preventing cervical cancers and pre-cancerous lesions, HPV vaccination can reduce costs associated with borderline and mild dysplasia, and associated colposcopies. Primary prevention of cervical cancer by vaccination against high-risk HPV types has been an important development for public health. Currently, licensed HPV vaccines protect against HPV types that cause approximately 70% of cervical cancers. Results from a phase III clinical trial of a new nonavalent HPV vaccine are now available. This vaccine has the potential to expand the prophylactic cervical cancer coverage offered by current HPV vaccines from approximately 70 to 90% and the prevention of high-grade dysplasia from approximately 50 to 75–85%. (52). First cost-effectiveness analyses conducted in Canada concluded that the nonavalent vaccine will most likely represent a cost-effective alternative to the quadrivalent vaccine (53).

## Answering unmet medical needs

In addition to the direct impact on healthcare resources and costs, vaccines can improve the sustainability of healthcare systems by answering unmet medical needs. The herpes zoster vaccine is a good example, since there are no preventive measures or satisfactory treatments. Nearly 1.7 million cases of zoster are diagnosed in Europe each year (54). The management of herpes zoster, and its most frequent complication, that is, PHN, a chronic neuropathic pain that can persist for months or even years, is challenging, particularly in elderly people suffering from other chronic diseases and on polypharmacy (55). The direct costs of zoster and PHN for the healthcare service have been estimated to be more than €60 million per year in a country such as France, and these costs can be expected to increase with the steadily growing elderly population (56). This economic burden is likely to be underestimated as the estimate did not include additional costs for society, such as, loss of productivity, treatment for worsening underlying conditions, home help, and rehabilitation care (54).

## Conclusion

Although the access to some new vaccines is heterogeneous across Europe, they have been shown to reduce healthcare costs associated with hospitalisations and outpatient visits in several real life impact studies. Prevention of disease in children, adults, and the elderly through vaccination represents a unique opportunity to keep people healthy and outside of the healthcare system. Hence, vaccination can contribute to the sustainability of healthcare systems by avoiding unnecessary use of financial and human resources and freeing resources for other medical interventions. Improving uptake of vaccination programmes is critical in periods when governments are looking for solutions for more efficient healthcare resource use. Thus, widespread health promotion and disease prevention are key factors for the long-term sustainability of health systems.

## References

[1] Whitney CG, Zhou F, Singleton J, Schuchat A (2014). Benefits from immunization during the vaccines for children program era – United States, 1994–2013. Morb Mortal Wkly Rep.

[2] Remy V, Zoellner Y, Heckmann U (2015). Vaccination: The cornerstone of an efficient healthcare system. J Mark Access Health Policy.

[3] Soriano-Gabarro M, Mrukowicz J, Vesikari T, Verstraeten T (2006). Burden of rotavirus disease in European Union countries. Pediatr Infect Dis J.

[4] Harris JP, Jit M, Cooper D, Edmunds WJ (2007). Evaluating rotavirus vaccination in England and Wales. Part I. Estimating the burden of disease. Vaccine.

[5] Giaquinto C, Van Damme P, Huet F, Gothefors L, Van der Wielen M (2007). Costs of community-acquired paediatric rotavirus gastroenteritis in 7 European countries: The REVEAL Study. J Infect Dis.

[6] Huet F, Largeron N, Trichard M, Miadi-Fargier H, Jasso-Mosqueda G (2007). Burden of paediatric rotavirus gastroenteritis and potential benefits of a universal rotavirus vaccination programme with RotaTeq in France. Vaccine.

[7] Public Health England (2014). Meningococcal disease: Guidance, data and analysis.

[8] Trotter CL, Edmunds WJ (2002). Modelling cost effectiveness of meningococcal serogroup C conjugate vaccination campaign in England and Wales. BMJ.

[9] Antonova EN, Rycroft CE, Ambrose CS, Heikkinen T, Principi N (2012). Burden of paediatric influenza in Western Europe: A systematic review. BMC Public Health.

[10] Kaye P, Malkani R, Martin S, Slack M, Trotter C, Jit M (2010). Invasive pneumococcal disease (IPD) in England & Wales after 7-valent conjugate vaccine (PCV7): Potential impact of 10 and 13-valent vaccines.

[11] Jiang Y, Gauthier A, Keeping S, Carroll S (2014). Cost-effectiveness of vaccinating the elderly and at-risk adults with the 23-valent pneumococcal polysaccharide vaccine or 13-valent pneumococcal conjugate vaccine in the UK. Expert Rev Pharmacoecon Outcomes Res.

[12] Health Protection Services UK (2014). Pneumococcal disease: Guidance, data and analysis.

[13] Jit M, Brisson M, Laprise JF, Choi YH (2015). Comparison of two dose and three dose human papillomavirus vaccine schedules: Cost effectiveness analysis based on transmission model. BMJ.

[14] Brotons P, Gelabert G, Launes C, Sicuri E, Pallares R, Munoz-Almagro C (2013). Cost of hospitalizing children with invasive pneumococcal pneumonia. Vaccine.

[15] Hartwig S, Baldauf JJ, Dominiak-Felden G, Simondon F, Alemany L, De Sanjose S Burden of HPV related cancers and precancerous lesions in women and men in Europe: Potential additional benefit of a 9-valent compared to the 4-valent vaccine in Europe (Abstract: PH.PP02.103).

[16] England PH (2014). Impact of first infant vaccination programme in England for rotavirus confirmed.

[17] Coudeville L, Brunot A, Giaquinto C, Lucioni C, Dervaux B (2004). Varicella vaccination in Italy: An economic evaluation of different scenarios. Pharmacoeconomics.

[18] ECDC (2014). Varicella vaccine in the European Union.

[19] Hartwig S, Syrjanen S, Dominiak-Felden G, Brotons M, Castellsague X (2012). Estimation of the epidemiological burden of human papillomavirus-related cancers and non-malignant diseases in men in Europe: A review. BMC Cancer.

[20] Raymakers AJ, Sadatsafavi M, Marra F, Marra CA (2012). Economic and humanistic burden of external genital warts. Pharmacoeconomics.

[21] Monsonego J, Breugelmans JG, Bouee S, Lafuma A, Benard S, Remy V (2007). Anogenital warts incidence, medical management and costs in women consulting gynaecologists in France. Gynecol Obstet Fertil.

[22] Ali H, Donovan B, Wand H, Read TR, Regan DG, Grulich AE (2013). Genital warts in young Australians five years into national human papillomavirus vaccination programme: National surveillance data. BMJ.

[23] Carroll S, Glenngard A, García Rojas AJ, Marin C (2015). Vaccination: Short- and long-term benefits from investment. J Mark Access Health Policy.

[24] Millier A, Aballea S, Annemans L, Toumi M, Quilici S (2012). A critical literature review of health economic evaluations in pertussis booster vaccination. Expert Rev Pharmacoecon Outcomes Res.

[25] Preaud E, Durand L, Macabeo B, Farkas N, Sloesen B, Palache A (2014). Annual public health and economic benefits of seasonal influenza vaccination: A European estimate. BMC Public Health.

[26] Council of the European Union (2009). Council recommendation of 22 December 2009 on seasonal influenza vaccination (text with EEA relevance) (2009/1019/EU).

[27] WHO (2014). Influenza (Seasonal) Fact sheet No. 211.

[28] Hannoun C (2013). The evolving history of influenza viruses and influenza vaccines. Expert Rev Vaccines.

[29] Reed C, Meltzer MI, Finelli L, Fiore A (2012). Public health impact of including two lineages of influenza B in a quadrivalent seasonal influenza vaccine. Vaccine.

[30] Lee BY, Bartsch SM, Willig AM (2012). The economic value of a quadrivalent versus trivalent influenza vaccine. Vaccine.

[31] Mc Laughlin J, Mc Ginnis JJ, TTan L, Mercatante A, Fortuna J (2015). Estimated human and economic burden of 4 major adult vaccine-preventable diseases in the US, 2013. J Prim Prev.

[32] van Seventer R, Sadosky A, Lucero M, Dukes E (2006). A cross-sectional survey of health state impairment and treatment patterns in patients with postherpetic neuralgia. Age Ageing.

[33] Charfi R, El Aidli S, Zaiem A, Kastalli S, Srairi S, Daghfous R (2012). Adverse drug reactions in older adults: A retrospective study from pharmacovigilance. Therapie.

[34] Bonanni P, Picazo J, Remy V (2015). The intangible benefits of vaccination – What is the true economic value of vaccination?. J Market Access Health Policy.

[35] Gleizes O, Desselberger U, Tatochenko V, Rodrigo C, Salman N, Mezner Z (2006). Nosocomial rotavirus infection in European countries: A review of the epidemiology, severity and economic burden of hospital-acquired rotavirus disease. Pediatr Infect Dis J.

[36] Ogilvie I, Khoury H, Goetghebeur MM, El Khoury AC, Giaquinto C (2012). Burden of community-acquired and nosocomial rotavirus gastroenteritis in the paediatric population of Western Europe: A scoping review. BMC Infect Dis.

[37] Festini F, Cocchi P, Mambretti D, Tagliabue B, Carotti M, Ciofi D (2010). Nosocomial rotavirus gastroenteritis in paediatric patients: A multi-center prospective cohort study. BMC Infect Dis.

[38] Pockett RD, Campbell D, Carroll S, Rajoriya F, Adlard N (2013). Rotavirus, respiratory syncytial virus and non-rotaviral gastroenteritis analysis of hospital readmissions in England and Wales. Acta Paediatr.

[39] Bouza E (2012). Consequences of *Clostridium difficile* infection: Understanding the healthcare burden. Clin Microbiol Infect.

[40] Kuijper EJ, Coignard B, Tull P (2006). Emergence of *Clostridium difficile*-associated disease in North America and Europe. Clin Microbiol Infect.

[41] Carman WF, Elder AG, Wallace LA, McAulay K, Walker A, Murray GD (2000). Effects of influenza vaccination of health-care workers on mortality of elderly people in long-term care: A randomised controlled trial. Lancet.

[42] Burls A, Jordan R, Barton P, Olowokure B, Wake B, Albon E (2006). Vaccinating healthcare workers against influenza to protect the vulnerable – Is it a good use of healthcare resources? A systematic review of the evidence and an economic evaluation. Vaccine.

[43] Bray F, Ren JS, Masuyer E, Ferlay J (2013). Estimates of global cancer prevalence for 27 sites in the adult population in 2008. Int J Cancer.

[44] IARC (2014). European code against cancer.

[45] Kane MA (2012). Preventing cancer with vaccines: Progress in the global control of cancer. Cancer Prev Res (Phila).

[46] Bosch FX, Broker TR, Forman D, Moscicki A-B, Gillison ML, Doorbar J (2013). Comprehensive control of human papillomavirus infections and related diseases. Vaccine.

[47] Borget I, Abramowitz L, Mathevet P (2011). Economic burden of HPV-related cancers in France. Vaccine.

[48] Preaud E, Largeron N (2013). Economic burden of non-cervical cancers attributable to human papillomavirus: A European scoping review. J Med Econ.

[49] Brown RE, Breugelmans JG, Theodoratou D, Benard S (2006). Costs of detection and treatment of cervical cancer, cervical dysplasia and genital warts in the UK. Curr Med Res Opin.

[50] Salter J (2014). Behind the screen.

[51] Dasbach E, Insinga R, Elbasha E (2008). The epidemiological and economic impact of a quadrivalent human papillomavirus vaccine (6/11/16/18) in the UK. BJOG.

[52] Chatterjee A (2014). The next generation of HPV vaccines: Nonavalent vaccine V503 on the horizon. Expert Rev Vaccines.

[53] Drolet M, Laprise JF, Boily MC, Franco EL, Brisson M (2014). Potential cost-effectiveness of the nonavalent human papillomavirus (HPV) vaccine. Int J Cancer.

[54] Gater A, Uhart M, McCool R, Preaud E (2015). The humanistic, economic and societal burden of Herpes Zoster in Europe: A critical review. BMC Public Health.

[55] Pickering G, Bertrand I, Marcoux M, Morel V, David L, Macian N Treatment of postherpetic neuralgia: A touchy mission in elderly patients, particularly in those with chronic diseases and polypharmacy (abstract: PP16).

[56] Mick G, Gallais JL, Simon F, Pinchinat S, Bloch K, Beillat M (2010). Burden of herpes zoster and postherpetic neuralgia: Incidence, proportion, and associated costs in the French population aged 50 or over. Rev Epidemiol Sante Publique.

# Sub article: Role of vaccination in economic growth

**DOI:** 10.3402/jmahp.v3.27044

**Published:** 2015-08-12

**Authors:** Sibilia Quilici, Richard Smith, Carlo Signorelli

**Affiliations:** 1 Sanofi Pasteur MSD, Lyon, France; 2 Faculty of Public Health and Policy, London School of Hygiene & Tropical Medicine, London, UK; 3 Department of Biomedical, Biotechnological & Translational Sciences (S.Bi.Bi.T), Public Health Unit, University of Parma, Parma, Italy

**Keywords:** Vaccination, macroeconomic, productivity, economic growth, investment

## Abstract

The health of a population is important from a public health and economic perspective as healthy individuals contribute to economic growth. Vaccination has the potential to contribute substantially to improving population health and thereby economic growth. Childhood vaccination programmes in Europe can offer protection against 15 important infectious diseases, thus preventing child fatalities and any serious temporary and permanent sequelae that can occur. Healthy children are more able to participate in education, thus preparing them to become healthy and productive adults. Vaccination programmes can also prevent infectious diseases in adolescents, thus allowing them to continue their development towards a healthy adulthood. Protecting adults against infectious diseases ensures that they can fully contribute to productivity and economic development by avoiding sick leave and lower productivity. Vaccination in older adults will contribute to the promotion of healthy ageing, enabling them to assist their familiy with, for instance, childcare, and also help them avoid functional decline and the related impacts on health and welfare expenditure. Effective vaccination programmes for all ages in Europe will thus contribute to the European Union's 2020 health and economic strategies. Indeed, beyond their impact on healthcare resources and productivity, reductions in mortality and morbidity also contribute to increased consumption and gross domestic product. Therefore, assessment of the value of vaccines and vaccination needs to consider not just the direct impact on health and healthcare but also the wider impact on economic growth, which requires a macroeconomic analysis of vaccination programmes.

The European financial and economic crisis, which started in 2008, has put pressure on healthcare budgets, resulting in cuts that are often short term and leading to unprecedented consequences on healthcare systems and the health of European citizens (1). In times of economic crisis and austerity, investments in health may need to be frozen. However, since health is closely linked to productivity and therefore the economic viability of individuals, populations, and nations, this may not be the wisest long-term strategy. Good health drives higher incomes through a number of mechanisms: education, labour productivity, tax contributions, investment, and savings (2–4) (Fig. 1). A recent European study estimated that the return on investment for each dollar of government spending in health was $4 (5). It has been shown that population health can operate through multiple channels, contributing to economic growth, which can in turn generate additional resources to reinvest into health, generating a virtuous cycle. Healthy children tend to achieve better educationally and to have better cognitive function, healthy adults tend to work longer and more productively, and healthy populations tend to save more and to attract more foreign investment contributing to capital accumulation, job creation, and technological progress (6). It is generally thought, especially in developing countries, that higher incomes promote better health through improved nutrition, sanitation, adoption of healthy lifestyles, and the ability to purchase better, high-quality healthcare.

**Fig. 1:** Potential mechanism for the link between health and economic output and the roles of clean water, prevention programmes, including vaccination, and hygiene (2–4).

Health enhancement is largely dependent on prevention, which can be considered to be the first level of healthcare (2). The importance of prevention was recognised many centuries ago by Desiderius Erasmus with his famous quote ‘Prevention is better than cure’. The primary objective of prevention is to protect health by avoiding diseases (primary prevention) and stopping or slowing the progress of diseases in their earliest stages (secondary prevention). As such, vaccination is recognised by many, including the World Health Organization, as one of the most cost-effective (and often cost-saving) primary prevention measures to help protect individuals and to promote public health.

Prevention programmes, such as vaccination, are often considered to have only return in the long run; thus, in situations where governments are looking to cut expenditure, they will look for short-term return and stop investing in prevention programmes. However, health should not be seen as an expenditure, or consumption good, but as an investment (7). In addition to its effectiveness in reducing disease and mortality, the benefits of vaccination have usually been measured in terms of the averted costs of medical care (8, 9). Sometimes consideration is also made of the immediate productivity loss to patients (as a result of illness or death) and their carers (10). However, in the longer term, it has been suggested that vaccines can increase lifetime productivity due to improved physical capacity, cognition, and educational outcomes through increased school attendance (11). Reductions in mortality and morbidity also contribute to increased consumption and gross domestic product (GDP) (6). For example, preliminary research suggested that a 5-year improvement in life expectancy can translate into 0.3 to 0.5% increased annual growth (6).

A framework to present these broader benefits has already been proposed, using the example of haemophilus influenza type b (Hib) vaccination (Table 2) and other available vaccines (6, 12). In this article, we aim to review the role of lifelong vaccination in economic growth, especially in Europe, and the need for a broader macroeconomic assessment framework for economic evaluations of vaccines.

## Paediatric vaccination programmes represent a long-term investment in future generations with a positive net present value for the society and the economy

Infant mortality rates (i.e., death of infants <1 year of age) reflect a country's economic and social conditions as well as the performance of its healthcare system. All European countries have achieved reductions in infant mortality rates since 1970, from an average of 25 deaths per 1,000 live births, to the current average of 4.2 per 1,000 births, corresponding to a cumulative reduction of over 80% (13). The reasons for this progress include improvements in sanitation, access to vaccination against infectious diseases, other public health measures, and wider social factors.

Investing in children's health is an investment in tomorrow's society. Good health from prenatal life to adolescence is a resource for social and economic development. The burden of ill health and impaired development in children has a multitude of effects. Sick children make additional demands on their parents, which can have an impact on the family's earning potential and can have detrimental consequences for their siblings. For example, the results from a study in South Africa have shown a significant association between coverage of measles vaccination and the level of school-grade attainment in siblings, suggesting that 1 year of schooling could be gained for every six children vaccinated against measles (6). Sick children also represent a cost for health and welfare systems that can sometimes continue into adult life. Poor cognitive and social development for children can create a lifetime of disadvantage, the legacy of which is often passed onto future generations.

From birth, a child is at risk of developing many severe infectious diseases that, in the absence of effective vaccines, can have serious consequences on their survival likelihood, as well as their physical and cognitive development (Table 1) (14). In the European Union (EU), childhood vaccination programmes protect against up to 15 different infectious diseases that could potentially result in high impairment with a huge impact on future human capital development (15). These diseases are tuberculosis, rotavirus, diphtheria, tetanus, pertussis, poliomyelitis, Hib, hepatitis b, pneumococcal disease, meningococcal disease, measles, mumps, rubella, varicella, and influenza. The benefits of infant vaccination against many of these diseases are often not taken into account, from a social and macroeconomic viewpoint. However, in the absence of these vaccines, substantial numbers of children would die, and many of those who would survive could develop mental and physical disabilities, preventing them from getting the most out of the education system and thereby impacting their productivity capacity when they reach adulthood (4). For example, up to 10% of Hib cases in children aged 2 months to 5 years are fatal, and up to 35% of the survivors can suffer from long-term permanent neurological sequelae such as deafness, blindness, mental retardation, epilepsy, and paralysis, which can affect their ability to go to school and to learn, which is in turn related to lower labour productivity and lifetime earnings that may result in less tax revenues and thus fewer resources available for public investments (Table 2) (12, 16).

**Table 1:** Potential medical impact of some vaccine-preventable infectious diseases (based on fact sheets available on the ECDC website) (14)

			Risk of		
			
Vaccine-preventable diseases	Age and population at risk of infection	Potential complications and medical impact	lifelong cognitive impairment	lifelong physical impairment	death
Measles	Can be contracted at any age	Pneumonia, encephalitis, death	x	x	x
Chickenpox	90% of cases in children aged <10 years. Fewer than 15% of chickenpox cases in people aged >15 years; most severe cases in adults, with chances of complications increasing with age	Encephalitis, secondary infections (severe streptococcus, skin infection), hepatitis, pneumonia: can be fatal in around 10% of cases	x	x	x
Pneumococcal disease	Any age but most likely to happen in children aged <2 years and adults aged >65 years	Bacterial meningitis, pneumonia, blood infection, septicaemia	x	x	x
Seasonal flu	Can be contracted at any age	Ear and sinus infections, pneumonia, heart inflammation, and death		x	x
Rotavirus gastroenteritis	Mostly in children aged <5 years	Severe dehydration (loss of 10% of weight in children), sometimes death			x
Whooping cough (pertussis)	Can be contracted at any age – most severe cases in babies <6 months of age	Coughing spells so bad that it is hard to eat, drink, or breathe. Can last for weeks and lead to pneumonia, seizures (jerking and staring spells), brain damage, or death	x	x	x
Hepatitis B	Chronic infection is most likely to develop in young babies. Most infections occur in adults in high-risk groups	Chronic infections can lead to inflammation of the liver, liver damage (called cirrhosis), and cancer		x	x
*Haemophilus influenza* type b (Hib)	Aged 2 months–5 years	Most common cause of bacterial meningitis in children before the introduction of the vaccination, leading to brain damage or death (up to 10% of cases)	x	x	x
Tetanus	The highest tetanus risk in Europe is found in the unvaccinated elderly	Painful tightening of muscles can lead to spasm, and death in 10% of cases		x	x
Polio	Can be contracted at any age	In children aged <5 years: paralysis of one leg is most common In adults: extensive paralysis of the chest and abdomen is more likely May lead to death		x	x
Diphtheria	Can be contracted at any age	Can lead to breathing problems, paralysis, heart failure, and even death		x	x
Meningococcal disease	Most frequently occurs in young children, but a second disease peak is observed among adolescents and young adults	Even when the disease is diagnosed early and adequate treatment is started, 5 to 10% of patients die, typically within 24 to 48 hours after the onset of symptoms. Bacterial meningitis may result in brain damage, hearing loss, or a learning disability in 10 to 20% of survivors	x	x	x
Mumps	Children aged 5–9 years most often affected	Deafness, meningitis (infection of the brain and spinal cord covering), painful swelling of the testicles or ovaries, and, rarely, death	x	x	x
Rubella	Children aged 4–9years most often affected	In women: arthritis, risks of miscarriage, congenital anomaly (deaf, blind, mentally retarded, or with heart or brain damage)	x	x	
Human papillomavirus (HPV)	Genital warts and HPV-related cancer: adolescents and young adults aged 16–25years	Precancerous cervical, vulvar, and vaginal lesions; cervical, vulvar, and vaginal cancer; genital warts		x	x

From Ref. (14).

**Table 2:** Type of benefits in economic evaluations of vaccinations: application to Hib vaccination

Perspective	Benefit categories	Definition	Hib-specific examples
Narrow	Health gains	Reduction in mortality through vaccination	Hundreds of thousands of children die each year from Hib disease
	Healthcare cost savings	Medical expenditure savings because vaccination prevents disease episodes	Hib disease leads to substantial healthcare costs
	Care-related productivity gains	Savings of parents’ productive time because vaccination avoids the need for missing work to take care of a sick child	Parental care of children suffering from Hib disease can contribute substantially to the overall cost of the disease
Broad	Outcome-related productivity gains	Increased productivity because vaccination improves cognition and physical strength, as well as school enrolment, attendance, and attainment	Hib meningitis is relatively common and leaves 15–35% of survivors with permanent disabilities, such as mental retardation or deafness, which can severely reduce cognition
	Behaviour-related productivity gains	Benefits accrue because vaccination improves child health and survival, and thereby changes household choices, such as fertility and consumption choices	Hundreds of thousands of children die each year from Hib disease
	Community externalities	Benefits accrue because vaccination improves outcomes among unvaccinated community members	Hib infections are treated with antibiotics, leading to the development of resistance. Hib vaccination can protect unvaccinated individuals through herd effects

From Ref. (12).

Other childhood diseases, such as mumps and varicella, can lead to serious complications, such as meningitis, and even death in adulthood. Childhood vaccination not only protects infants and young children from these debilitating diseases but also can provide protection to adults and the elderly through prevention of transmission from the younger individuals (12, 17).

## Medium- and long-term investment in adolescent and young adult vaccination programmes

Adolescents and young adults are at risk of many infectious diseases such as pertussis, meningococcal meningitis, as well as sexually transmissible diseases caused by pathogens such as hepatitis B or human papillomavirus (HPV). The consequences of an outbreak of measles in 2008–2011 in France highlighted that adolescents and young adults are particularly vulnerable to this disease (18). These infections can lead to short- and medium-term complications (e.g., severe cough from pertussis, brain damage from meningitis, and genital warts from HPV), as well as a long-term risk to develop HPV-related cancers and chronic liver disease from hepatitis B (Table 1). These complications all have substantial consequences on the social and economic activities of these populations. For example, it has been reported that when a member of a household had cervical cancer, behaviour such as daily food consumption and school attendance could change, and this could negatively impact both their educational attainment and earnings (6, 19, 20).

Thus, adolescent and young adult vaccination, through boosters or catch-up programmes, provides medium- and long-term return on public health investment by protecting this population from debilitating diseases that can impact their development prior to adulthood.

## Rapid health and productivity gain from adult vaccination programmes

Infectious diseases in adulthood can cause substantial disruption for family and professional activities with a cumulative economic impact when considered on a national scale. For example, the whole population is susceptible to influenza every year, although the extent of the risk and the consequences are dependent on the circulating strains. A professionally active person who has influenza-like illness will take, on average, 2 to 5 days of sick leave (21). When this is multiplied by the number of working individuals infected in different economic sectors, there is a substantial impact on the economic growth of a nation. Sick days are a burden to individuals as they can represent a considerable proportion of their earnings, but they also result in substantial losses for the firm, which reduces its profitability. In France, for example, it has been estimated that working adults had to stop working for a mean of 4 days for influenza (21, 22). In Norway, the mean number of working days lost for seasonal influenza annually was estimated to be 793,000, resulting in an estimated productivity loss of US$231 million (23). This is without taking into account the decline in economic activity due to mass working force absenteeism or the reduced productivity while at work (presenteeism), which may pose a substantial economic burden on firms due to the loss of productive output.

Another vaccine-preventable disease in adulthood with potential consequences on work productivity is herpes zoster (HZ), more commonly known as shingles. HZ arises from the reactivation of a varicella virus that remains dormant after a childhood episode of varicella. The reactivation can be caused by a number of factors, including a decline of the immune system with age. HZ can lead to debilitating pain-related complications, such as post-herpetic neuralgia (PHN). In a recent study, it was reported that two-thirds of working adults (aged 50–65) who had HZ stopped working and about 75% reported decreased effectiveness at work (i.e., presenteeism) during almost 2 days because of HZ or PHN (24). Maintaining a healthy and productive workforce is a key priority for public debt sustainability and economic growth. Vaccination programmes can also protect patients with chronic conditions, thereby leading to substantial economic benefits for those who are working, which contributes to the economy and government tax base. Patients with chronic diseases, such as diabetes or chronic heart disease, are at higher risk of infectious diseases (25). For instance, patients with diabetes, who represent about 10% of the population aged 25 years and over in Europe (26), are at higher risk of HZ than individuals without diabetes, as was observed in a recent US study in which diabetes was associated with 45 and 18% adjusted risks for HZ and PHN, respectively (27). A recent European study also demonstrated that people with underlying conditions accounted for the greatest share of total costs avoided due to influenza vaccination, as most of these people are productively employed (28). Thus, vaccination programmes, such as influenza or HZ, can offer a clear contribution to improving economic productivity and minimising workforce absenteeism by preventing infection and diseases, in particular in those with chronic conditions (10).

## Elderly vaccination programmes contribute to a more active and healthier ageing population in a context of unprecedented demographic challenge in Europe

The global population is ageing, and Europe is by far the oldest continent. After Japan, Germany and Italy have the 2nd and 3rd highest median ages in the world (29). It is estimated that the population aged ≥65 years will almost double from 87.5 million in 2010 to 152.6 million in 2060. The demographic old-age dependency ratio (i.e., the ratio of the number of people aged ≥65 years to those aged 15–64 years) is projected to increase from 26% in 2010 to 52% in 2060 in the EU (30). This means that, by 2060, the number of working-age people supporting each pensioner will drop by half, not only making state pensions harder to afford but also raising the question of who will take care of this ageing population from a health and social perspective. Thus, the changing demographic situation is a serious threat to the financial and social-economic sustainability of the EU member states. Ensuring the ageing society will remain independent for longer and continue contributing to the economy and to society is key. Therefore, rapid demographic ageing is not only a major societal challenge (in terms of public budgets, workforce, competitiveness, and quality of life) but also a major opportunity for new jobs and growth, also referred to as the *Silver Economy* (31).

We can understand why the elderly are at higher risk of infectious diseases due to their declining immune system (immunosenescence) and their higher risk of comorbidities (32). They also have a higher risk of severe outcomes from infections because these are also strongly associated with unhealthy lifestyle, dietary deficiency, and polymedication (33). Very common infectious diseases in the elderly, such as influenza, pneumococcal infections, and HZ, can lead to complications that may contribute to or accelerate their overall functional decline, sometimes leading even to death. When elderly patients are hospitalised, they often experience reduced mobility and activity levels, increasing their risk for functional decline and dependency. Keeping this population away from hospitals should contribute to keeping them active and healthier and, therefore, not only less dependent but also able to assist their wider family with, for instance, childcare (10, 34).

For example, the results from a study in the United States demonstrated the substantial effectiveness of influenza vaccination and its benefits to healthcare systems (35). Influenza vaccination for the 2010–2011 season prevented more than 75% of adult hospitalisation in those aged over 50 years (35). Vaccination, as a key element in a primary prevention strategy against influenza, pneumococcal diseases, and shingles, can thus play a crucial role in keeping the ageing population active and healthy.

## Macroeconomic impact of vaccination

Investment in vaccination thus offers a wide range of economic and intangible benefits that can potentiate gains for the individual and for society (10, 36). As such, vaccination not only is a healthcare sector issue but also has repercussions for wider economic planning and long-term economic progress. However, the conventional economic evaluations usually conducted for vaccination generally omit health-related productivity and macroeconomic improvements attributed to health status changes and, consequently, may not adequately reflect the broader economic benefits of vaccination. The scope of macroeconomic evaluation is quite different from the microeconomic assessments that are widely used in cost-effectiveness analyses for drug regimens. For vaccination, the focus is to capture in addition the rate of return of public health investment (37). For example, the results from one study demonstrated that the net benefit of 60 years of investment in polio vaccine was six-fold higher (approximately $180 billion) than the cost during that time (16). This estimated positive net benefit was essentially based on the savings in treatment costs without taking into account the intangible costs of productivity loss and death. Hence, additional methods should be considered to capture the full benefits of vaccination, such as assessment of vaccination's impact on absenteeism, presenteeism, or individuals’ lifetime earnings due to improved educational achievement.

This approach has been used to evaluate the potential macroeconomic impact of pandemic influenza in the United Kingdom, including associated behavioural responses, school closure, and vaccination (38). The costs related to influenza alone ranged from between 0.5 and 1.0% of the GDP (between £8.4bn and £16.8bn) for high-fatality scenarios, and larger still for an extreme pandemic scenario. It was shown that vaccination with a pre-pandemic vaccine could save from 0.13 to 2.3% of the GDP (between £2.2bn and £38.6bn) over a single year (Fig. 2).

**Fig. 2:** Effect of pandemic influenza on UK gross domestic product (GDP) according to various disease and mitigation scenarios (all strategies assumed to a 60% vaccine uptake) (38).

Other studies have estimated how lives saved could influence future government expenditure on social programmes such as health, education, and pensions, as well as influence future tax receipts. This is referred to as the ‘government perspective’ analysis and requires constructing a model that reflects the life course of average citizens, taking into consideration, for example, average schooling, employment, marriage, wages, and pension costs. Using this approach, a study conducted in Egypt concluded that investment costs in rotavirus vaccination for a cohort of infants would be entirely offset when the vaccinated cohort were 22 years old (39). Another study estimated the governmental return on investment for immunising adults aged 50 against diphtheria, tetanus, pertussis, seasonal influenza, pneumococcal diseases, and HZ in the Netherlands, by considering how investments in immunisation influence ongoing tax revenues to government (e.g., income tax, value-added tax, and social insurance contributions). Based on the investment costs of vaccinating adults aged 50, vaccination yielded a benefit–cost ratio of 4.09, suggesting a fourfold rate of return for the government (40).

Public investments in vaccination can therefore provide a significant public health benefit that translates into return on investment attributed to reduced government expenditure and increased tax revenues.

## Conclusions

Health is a key factor for the promotion of economic growth at the national, regional, and global levels. The vaccine industry and vaccination programmes targeted at populations of different ages can contribute substantially to economic growth by keeping people healthy throughout their lives, with continuous investment in research & development to protect populations against an increasing number of existing or new vaccine-preventable diseases. There is a clear need for a commitment to vaccination not only from health authorities but also from governments. In particular, the finance ministries and treasuries of different governments need to assess how best vaccines and vaccination can make an efficient contribution to their national economic growth, and thus to European growth. As such, macroeconomic analyses offer a critical evaluation tool but are rarely used. Greater impetus and investment in their use is needed to provide evidence to determine the full economic value of vaccination.

## References

[1] Mladovsky P, Srivastava D, Cylus J, Karanikolos M, Evetovits T, Thomson S Health policy responses to the financial crisis in Europe 2012.

[2] Loeppke R, Nicholson S, Taitel M, Sweeney M, Haufle V, Kessler RC (2008). The impact of an integrated population health enhancement and disease management program on employee health risk, health conditions, and productivity. Popul Health Manag.

[3] Suhrcke M, McKee M, Stuckler D, Sauto Arce R, Tsolova S, Mortensen J (2006). The contribution of health to the economy in the European Union. Public Health.

[4] Bloom DE, Canning D, Weston M (2005). The value of vaccination. World Econ.

[5] Reeves A, Basu S, McKee M, Meissner C, Stuckler D (2013). Does investment in the health sector promote or inhibit economic growth?. Global Health.

[6] Barnighausen T, Bloom DE, Cafiero-Fonseca ET, O'Brien JC (2014). Valuing vaccination. Proc Natl Acad Sci USA.

[7] Suhrcke M, Stuckler D, Suk JE, Desai M, Senek M, McKee M (2011). The impact of economic crises on communicable disease transmission and control: A systematic review of the evidence. PLoS One.

[8] Rémy V, Zöellner Y, Heckmann U (2015). Vaccination: The cornerstone of an efficient healthcare system. J Market Access Health Policy.

[9] Largeron N, Lévy P, Wasem J, Bresse X (2015). Role of vaccination in healthcare systems sustainability. J Market Access Health Policy.

[10] Postma M, Carroll S, Brandão A (2015). The societal impact of direct and indirect protection from lifespan vaccination. http://dx.doi.org/10.3402/jmahp.v3.26962.

[11] Deogaonkar R, Hutubessy R, van der Putten I, Evers S, Jit M (2012). Systematic review of studies evaluating the broader economic impact of vaccination in low and middle income countries. BMC Public Health.

[12] Barnighausen T, Bloom DE, Canning D, Friedman A, Levine OS, O'Brien J (2011). Rethinking the benefits and costs of childhood vaccination: The example of the Haemophilus influenzae type b vaccine. Vaccine.

[13] OECD (2012). Infant mortality. Health at a glance: Europe 2012.

[14] ECDC (2014). Health topics A–Z.

[15] ECDC Vaccine schedule 2014.

[16] ECDC Haemophilus influenzae: Factsheet for health professionals 2014.

[17] Thompson KM, Tebbens RJ (2006). Retrospective cost-effectiveness analyses for polio vaccination in the United States. Risk Anal.

[18] Antona D, Levy-Bruhl D, Baudon C, Freymuth F, Lamy M, Maine C (2013). Measles elimination efforts and 2008–2011 outbreak, France. Emerg Infect Dis.

[19] Arrossi S, Matos E, Zengarini N, Roth B, Sankaranayananan R, Parkin M (2007). The socio-economic impact of cervical cancer on patients and their families in Argentina, and its influence on radiotherapy compliance. Results from a cross-sectional study. Gynecol Oncol.

[20] Barnighausen T, Bloom DE, Cafiero ET, O'Brien JC (2012). Economic evaluation of vaccination: Capturing the full benefits, with an application to human papillomavirus. Clin Microbiol Infect.

[21] Keech M, Beardsworth P (2008). The impact of influenza on working days lost: A review of the literature. Pharmacoeconomics.

[22] Carrat F, Sahler C, Rogez S, Leruez-Ville M, Freymuth F, Le Gales C (2002). Influenza burden of illness: Estimates from a national prospective survey of household contacts in France. Arch Intern Med.

[23] Xue Y, Kristiansen IS, de Blasio BF (2010). Modeling the cost of influenza: The impact of missing costs of unreported complications and sick leave. BMC Public Health.

[24] Drolet M, Levin MJ, Schmader KE, Johnson R, Oxman MN, Patrick D (2012). Employment related productivity loss associated with herpes zoster and postherpetic neuralgia: A 6-month prospective study. Vaccine.

[25] Shah BR, Hux JE (2003). Quantifying the risk of infectious diseases for people with diabetes. Diabetes Care.

[26] World Health Organization (2010). The challenge of diabetes: Data and statistics.

[27] Suaya JA, Chen SY, Li Q, Burstin SJ, Levin MJ (2014). Incidence of herpes zoster and persistent post-zoster pain in adults with or without diabetes in the United States. Open Forum Infect Dis.

[28] Preaud E, Durand L, Macabeo B, Farkas N, Sloesen B, Palache A (2014). Annual public health and economic benefits of seasonal influenza vaccination: A European estimate. BMC Public Health.

[29] IndexMundi (2015). http://www.indexmundi.com.

[30] European Commission (2012). The 2012 ageing report: Economic and budgetary projections for the 27 EU Member States (2010–2060).

[31] Commission E (2015). Growing the European silver economy: Background paper.

[32] Heppner HJ, Cornel S, Peter W, Philipp B, Katrin S (2013). Infections in the elderly. Crit Care Clin.

[33] Aspinall R, Del Giudice G, Effros RB, Grubeck-Loebenstein B, Sambhara S (2007). Challenges for vaccination in the elderly. Immun Ageing.

[34] Kleinpell RM, Fletcher K, Jennings BM, Hughes RG (2008). Reducing functional decline in hospitalized elderly. Patient safety and quality: An evidence-based handbook for nurses.

[35] Talbot HK, Zhu Y, Chen Q, Williams JV, Thompson MG, Griffin MR (2013). Effectiveness of influenza vaccine for preventing laboratory-confirmed influenza hospitalizations in adults, 2011–2012 influenza season. Clin Infect Dis.

[36] Bonanni P, Picazo J, Rémy V (2015). The intangible benefits of vaccination – What is the true economic value of vaccination?. J Market Access Health Policy.

[37] Kotsopoulos N, Connolly MP (2014). Is the gap between micro- and macroeconomic assessments in health care well understood? The case of vaccination and potential remedies. J Market Access Health Policy.

[38] Smith RD, Keogh-Brown MR, Barnett T, Tait J (2009). The economy-wide impact of pandemic influenza on the UK: A computable general equilibrium modelling experiment. BMJ.

[39] Connolly MP, Topachevskyi O, Standaert B, Ortega O, Postma M (2012). The impact of rotavirus vaccination on discounted net tax revenue in Egypt: A government perspective analysis. Pharmacoeconomics.

[40] Kotsopoulos N, Connolly M Fiscal impact of adult vaccination in The Netherlands.

# Sub article: The societal role of lifelong vaccination

**DOI:** 10.3402/jmahp.v3.26962

**Published:** 2015-08-12

**Authors:** Maarten J. Postma, Stuart Carroll, Alexandra Brandão

**Affiliations:** 1 Unit of Pharmaco Epidemiology & Pharmaco Economics, Department of Pharmacy, University of Groningen, Groningen, Netherlands; 2 Institute of Science in Healthy Aging & Healthcare, University Medical Center Groningen, Groningen, Netherlands; 3 Sanofi Pasteur MSD, Maidenhead, United Kingdom; 4 Sanofi Pasteur MSD, Amadora, Portugal

**Keywords:** vaccination, quality of life, societal, indirect protection, productivity

## Abstract

The full economic and societal value of vaccination is complex to assess. Although direct protection is the immediate goal of vaccination programmes, it is rare that 100% uptake is attained. An important facet of vaccines value comes from the indirect (or herd) protection they provide. The evolving dynamics of our society, including the increase in the proportion of older individuals enhances the value of indirect protection in reducing disease transmission within the family setting and the society as a whole. For example, grandparents are increasingly involved in childcare, putting them at risk of disease transmission if they or the children are not vaccinated. Preventing disease in children can also reduce absenteeism for parents who otherwise would take days off work to care for their sick children, leading to a substantial societal burden. Preventing disease in working adults reduces absenteeism and presenteeism, enhancing productivity and contributing in turn to economic growth. Quality of life is essential at all ages. It is fundamental in children for their life chances, educational achievements, and healthy wellbeing. Additionally, preventing common diseases in adults and the elderly also contributes to their quality of life and helps to assure healthy ageing for growing ageing populations. These wider economic and societal values, although difficult to measure, should be taken into consideration in assessments of the economic value and cost-effectiveness of vaccination programmes.

Vaccination is often regarded as an individual intervention with a wider public health impact. By vaccinating one person, protection can be conferred to a wider group of people through the phenomenon of ‘herd effect’ that provides indirect protection. In today's highly globalised world, the demographic changes occurring and the increasing cross-border population movements and migration between countries and continents have important implications for global health. Controlling the spread of diseases, and related transmission dynamics, is a key challenge in global health, and the role and value of vaccination in this area are obvious. Vaccines have an important role for both preventing disease and reducing societal burden through the prevention of indirect costs of disease, such as absenteeism from work, productivity losses, and working days lost for parents and caregivers. In this article, we will present some examples of vaccination's indirect protection and its associated benefits within the family and the broader society, as well as examples of vaccination's impact on absenteeism, productivity and quality of life, which are of critical importance in the growing elderly populations.

## Enhancing value of vaccination through indirect protection

One of the unique properties of vaccination – and sometimes even potentially one of the main goals – is its ability to confer indirect protection through a herd effect to unvaccinated individuals and groups (1). Vaccination of a fraction of a population reduces the number of those susceptible to infection within the population and, thus, the probability of infection that can result in disease is also reduced. Hence, vaccination can control the transmission of the causative agent and limit the associated infection both directly and indirectly. This benefit is increasingly important given today's globalisation and nomadic cross-border population movements (2) and emphasises the broader value of vaccination as an essential preventative healthcare intervention.

The conjugate pneumococcal vaccine provides an example of the economic benefits of indirect protection. In Germany, the results from a cost-effectiveness study showed that the total costs from pneumoccal disease for the entire German birth cohort were €808.3 million without vaccination and €928.1 million with vaccination, when the benefits from herd effects were not considered (3). The incremental cost per life-year (LY) gained with vaccination was estimated to be over €100,000, that is, likely not cost-effective. However, when herd effect benefits were considered for the same German birth cohort, the total costs were €1,281.4 million with no vaccination and €1,288 million with vaccination. The incremental cost per LY gained with vaccination was below €200/LY gained, that is, highly cost-effective, almost cost-saving. Similar analyses have been performed in other countries, for example, the Netherlands (4). Excluding herd effects in economics evaluations of vaccines thus underestimates the value of vaccination by limiting the concept of value to direct effects only; this can lead to sub-optimal and inefficient public health decisions.

## The value of indirect protection within families and society

Vaccination protects and influences circumstances in whole families. Indirect protection is key if the impact that infectious diseases in children can have on families’ dynamics and caregivers’ absenteeism is considered. For example, a child with varicella may be excluded from school for up to 2 weeks, which corresponds to the disease's incubation period. When the child recovers and returns to school, infected siblings may exhibit symptomatic disease and be off school for another 2 weeks, prolonging parental or caregivers’ absenteeism. The importance of the role of grandparents is also highlighted by policies implemented in some European countries. In Germany, parents are entitled to take leave for up to 3 years after their child is born, 12 months of which can be deferred until the child is eight. Working grandparents may also take 10 days leave in an emergency to care for their grandchildren or to take unpaid leave of up to 6 months (5). In the UK, results from the Millennium Cohort Study showed that in 42% of families, 9-month-old children were looked after by grandparents when parents were at work, illustrating the importance of healthy grandparents also from this viewpoint (6). Changes in parental working patterns, linked with the current economic climate, have reinforced this trend. Therefore, indirect protection has become more important, due to these increasing contacts between these generations. Vaccination of children can reduce transmission to susceptible parents, and particularly older grandparents who can be more susceptible to infectious diseases (i.e., varicella, rubella, and pneumococcal disease) and have a higher risk of severe complications.

The role of indirect protection is also pertinent for wider protection across society, extending protection benefits to population groups that are not, cannot be or will only be reluctantly vaccinated (i.e., newborn infants, pregnant women, immunocompromised individuals). This protection may be crucial in public places, such as public transports, schools, and workplaces. The dynamic nature of mixing and contact patterns within a given population is a strong argument in favour of vaccination to provide both aggregate direct, and subsequent indirect protection to reduce disease transmission within the society as a whole. Even if some of the broader benefits of indirect protection may be difficult to quantify in monetary terms and, thus, challenging to include in economic evaluations (7), public health workers across European countries need to recognise and evaluate these societal benefits to inform policy decisions concerning vaccination and competing alternatives.

## Reducing the societal and caregiver burden

It is increasingly acknowledged that the costs of disease not only fall on the individual patient but also on caregivers including family, friends, communities, and the wider society. It is from this perspective that vaccination against preventable infectious diseases warrants a broader societal value. As mentioned previously, varicella disease in children incurs considerable indirect costs as a result of parental absenteeism and loss of productivity (8, 9). For example, the total indirect costs of varicella over a 50-year period in Italy were estimated to be €2,280 million (10). In Germany, the total annual costs of varicella for payers was estimated to be €78 million, the largest portion of which was due to the significant work loss costs incurred by parents caring for their sick children. For the society, the total annual costs were estimated to be €187.5 million, 82% of which corresponded to indirect costs (11).

Another study conducted in seven European Union (EU) countries estimated the percentage of rotavirus gastroenteritis (RVGE) cases that required at least one parent or another person to be absent from work was up to 91% for hospitalised children, between 4 and 64% for those attending emergency departments and between 20 and 64% for those seen in primary care (12). Similarly, a review conducted in Western Europe showed that between 11 and 61% of parents of children with influenza took leave from work for their own influenza infection or to take care of their children for a mean period of between 1.3 and 6.3 days (13). Bearing the average daily salary in EU countries in mind [median gross hourly earnings of €12 in EU-27, up to €25 in Denmark in 2010 (14)], the example of absenteeism generated by rotavirus and influenza infections equates to a significant indirect costs. In France, the economic burden of productivity loss represents almost 50% of the total cost of RVGE (15). Rotavirus vaccines have demonstrated a high efficacy in reducing the number of work days lost for parents to take care for their children and could thereby reduce this societal burden (16). Lastly, other additional productivity losses and costs associated with replacing staff, social security, or other health insurance payments should be considered. These aspects obviously further strengthen the economic value of vaccination in reducing indirect costs from a societal perspective.

## Minimising workforce absenteeism and improving economic productivity

Absenteeism can have a profound economical impact by undermining productivity in the workplace. For example, in the UK, each absent employee cost their employer an average of £975 in 2012, while absenteeism direct costs alone amounted to more than £14 billion a year across the economy (17). Loss of productivity is a key cost associated with absenteeism followed by the cost of payments for sickness leave and the cost of replacing staff to cover the absent employee. In addition, reduced productivity in the form of presenteeism is a consequence of illness at work and may potentially even outweigh the cost of absenteeism. For example, the total cost of presenteeism to the Australian economy was estimated to be AU$34.1 billion in 2009–2010, equating to a 2.7% decrease in the 2010 gross domestic product (18). The total cost of presenteeism to US employers ranges from about $150 to $250 billion annually, representing about 60% of the total cost of workers’ illness (19). Healthier people can not only work longer, they are also able to work more productively.

As an adjacent issue, vaccination of healthcare workers can help to improve the productivity of healthcare systems, where the level of absenteeism is becoming increasingly problematic, affecting the quality of care and resource management. This is especially pertinent in the case of influenza vaccination. Research suggests that even a 1% decrease in absenteeism of healthcare workers could lead to savings of around £34.2 million for the National Health Service in the UK (20). Additionally, in healthcare workers influenza and pertussis vaccination can provide crucial indirect protection to patients being cared for.

As populations grow older and the retirement age is increased to cope with the financial pressure of retirement funds of social security organisations, avoiding preventable disease in the working population and the ‘young’ elderly becomes more important. For example, herpes zoster (HZ) and post-herpetic neuralgia (PHN) have a negative impact on the productive work life of individuals. In a Canadian study, 64% of the employed participants reported missing work and 76% reported decreased effectiveness at work (i.e., presenteeism) due to HZ and PHN, for a mean number of 43 and 46 h, respectively (21). Vaccinating against HZ, pneumococcal disease, or flu could, therefore, help to contribute to healthy ageing, ensuring that people remain active, independent, and continue to be an asset for society. As described in another paper in this supplement, negative effects from absenteeism in working-aged adults go beyond the healthcare setting, affecting many industrial and service sectors (22). Reducing absenteeism from preventable disease and enhancing productivity are, therefore, essential for generating sustainable economic growth and making healthcare systems more affordable. Vaccination can strongly contribute to these societal challenges.

## Lifelong quality of life and healthy ageing

A central role of public health policy is to protect lives by reducing the burden of infectious diseases and preventing premature deaths. Vaccination has successfully contributed to these key goals. In addition, public health policy also aims to improve the quality of life and promote healthy ageing for all citizens.

European governments have recognised the importance of healthy ageing as part of the inevitable demographic changes occurring in many countries (22, 23). As populations age, there is an inevitable increase in individuals with chronic, long-term conditions. Preventing disease to foster healthy ageing is important, not just in terms of contributing to healthcare systems sustainability and affordability but also from the point of view that elderly individuals often constitute the most active group of volunteers in a society and are central to many community-based projects.

Also, in this context, vaccinations against influenza, pneumococcal disease, and HZ can be considered as quality-of-life enhancing interventions. A recent UK study that investigated the clinical presentation and quality-of-life burden of HZ and PHN from individual, clinical, and societal perspectives found that the pervasive nature of PHN pain and associated symptoms placed significant strains on individual and healthcare resources (24–26). The results also showed that the burden of disease extended beyond pain, with patients experiencing symptoms, such as emotional distress and depression, which all contributed to significant productivity losses. Notably, this societal value extends to the community as a whole. Preventing disease, although vitally important from an economic and employment perspective, is potentially even more fundamental to protecting and enhancing social, personal, and family activities.

## Socio-economic status, health, and missed equity

The principle of equity and equal access to maximise populations’ health is a keystone of modern healthcare systems. Absence of equity and equal access can result in significant missed opportunities, inflating social security and healthcare expenses with the well-known societal consequences that invariably occur. This, in turn, strengthens the adagium ‘prevention is better than cure’ as a general value proposition also as a means of reducing health inequalities. For example, a recent study in the UK showed that hospital admissions for all-cause gastroenteritis in children increased with a lower level of socio-economic status (27). The study concluded that the implementation of a rotavirus vaccination programme would help to reduce the burden of RVGE and all-cause gastroenteritis, and in this context, could have an impact on healthcare and social inequalities. Herd protection from vaccination may also play a role in indirectly protecting populations with lower socio-economic conditions, who may be harder to reach and have poorer access to healthcare and vaccination programmes. Finally, for example, HZ's complications and other infections in advanced age may lead to early retirement, impacting on retirement plans and potentially affecting socio-economic conditions of pensionados.

## Conclusions

Vaccination leads to lifelong individual and societal benefits, helping to reduce indirect costs, such as productivity losses and absenteeism from work, and improve quality of life. These key aspects of value contribute to equity and, in turn, avoid unequal access and health differences related to socio-economic status. Evaluations of vaccines should, therefore, consider these wider dimensions of value, particularly in the context of indirect protection. The societal benefits resulting from vaccination, although difficult to ascertain, should not be underestimated; they are fundamental to the true value proposition of vaccination. Wider social value, in addition to economic value, should be captured as part of routine assessment of the economic interest and cost-effectiveness of vaccination programmes.

## References

[1] Beutels P (2002). Economic evaluation of vaccination programmes in humans: A methodological exploration with application to hepatitis B, varicella-zoster, measles, pertussis, hepatitis A and pneumococcal vaccination.

[2] Ehreth J (2003). The global value of vaccination. Vaccine.

[3] Lloyd A, Patel N, Scott DA, Runge C, Claes C, Rose M (2008). Cost-effectiveness of heptavalent conjugate pneumococcal vaccine (Prevenar) in Germany: Considering a high-risk population and herd immunity effects. Eur J Health Econ.

[4] Rozenbaum MH, Sanders EA, van Hoek AJ, Jansen AG, van der Ende A, van den Dobbelsteen G (2010). Cost effectiveness of pneumococcal vaccination among Dutch infants: Economic analysis of the seven valent pneumococcal conjugated vaccine and forecast for the 10 valent and 13 valent vaccines. BMJ.

[5] Glaser K, Montserrat ER, Waginger U, Price D, Stuchbury R, Tinker A Grandparenting in Europe and the US.

[6] Dex S, Joshi H Millennium Cohort Study first survey: A user's guide to initial findings.

[7] Szucs T, Quilici S, Panfilo M (2015). From population to public institutions: What needs to be changed to benefit from the full value of vaccination. J Market Access Health Policy.

[8] Bonanni P, Boccalini S, Bechini A, Banz K (2008). Economic evaluation of varicella vaccination in Italian children and adolescents according to different intervention strategies: The burden of uncomplicated hospitalised cases. Vaccine.

[9] De Wals P, Blackburn M, Guay M, Bravo G, Blanchette D, Douville-Fradet M (2001). Burden of chickenpox on families: A study in Quebec. Can J Infect Dis.

[10] Coudeville L, Brunot A, Giaquinto C, Lucioni C, Dervaux B (2004). Varicella vaccination in Italy: An economic evaluation of different scenarios. Pharmacoeconomics.

[11] Banz K, Wagenpfeil S, Neiss A, Hammerschmidt T, Wutzler P (2004). The burden of varicella in Germany. Potential risks and economic impact. Eur J Health Econ.

[12] Van der Wielen M, Giaquinto C, Gothefors L, Huelsse C, Huet F, Littmann M (2010). Impact of community-acquired paediatric rotavirus gastroenteritis on family life: Data from the REVEAL study. BMC Fam Pract.

[13] Antonova EN, Rycroft CE, Ambrose CS, Heikkinen T, Principi N (2012). Burden of paediatric influenza in Western Europe: A systematic review. BMC Public Health.

[14] European Commission Eurostats: Wages and labour costs.

[15] Huet F, Largeron N, Trichard M, Miadi-Fargier H, Jasso-Mosqueda G (2007). Burden of paediatric rotavirus gastroenteritis and potential benefits of a universal rotavirus vaccination programme with RotaTeq in France. Vaccine.

[16] Vesikari T, Matson DO, Dennehy P, Van Damme P, Santosham M, Rodriguez Z (2006). Safety and efficacy of a pentavalent human-bovine (WC3) reassortant rotavirus vaccine. N Engl J Med.

[17] Confederation of British Industry (CBI) Fit for purpose. Absence and workplace health survey 2013.

[18] Medibank (2011). Sick at work. The cost of presenteeism to your business and the economy.

[19] Schaefer P The hidden costs of presenteeism: Causes and solutions.

[20] Walker V, Bamford D (2011). An empirical investigation into health sector absenteeism. Health Serv Manage Res.

[21] Drolet M, Levin MJ, Schmader KE, Johnson R, Oxman MN, Patrick D (2012). Employment related productivity loss associated with herpes zoster and postherpetic neuralgia: A 6-month prospective study. Vaccine.

[22] Quilici S, Smith R, Signorelli C (2015). The role of vaccination in economic growth. JMAPH.

[23] European Commission The 2012 ageing report.

[24] Carroll S, Gater A, Abetz L, Demuth D, Smith F, Mannan A (2012). Understanding disease presentation and patient-reported and economic impact of herpes zoster (HZ) and post-herpetic neuralgia (PHN): Findings from the Zoster Quality Of Life (ZQOL) Study (abstract: PSY34). Value Health.

[25] Gater A, Carroll S, Abetz L, Demuth D, Smith F, Mannan A (2010). Quantifying the patient-reported burden of herpes zoster and post-herpetic neuralgia in the UK: Practical considerations.

[26] Gater A, Carroll S, Abetz L, Demuth D, Smith F, Mannan A (2011). Burden of herpes zoster and post-herpetic neuralgia: Findings from a cross-sectional patient reported outcomes study.

[27] Pockett RD, Adlard N, Carroll S, Rajoriya F (2011). Paediatric hospital admissions for rotavirus gastroenteritis and infectious gastroenteritis of all causes in England: An analysis of correlation with deprivation. Curr Med Res Opin.

# Sub article: The intangible benefits of vaccination – what is the true economic value of vaccination?

**DOI:** 10.3402/jmahp.v3.26964

**Published:** 2015-08-12

**Authors:** Paolo Bonanni, Juan José Picazo, Vanessa Rémy

**Affiliations:** 1 Department of Health Sciences, University of Florence, Florence, Italy; 2 Department of Clinical Microbiology, Hospital Clinico San Carlos, Madrid, Spain; 3 Sanofi Pasteur MSD, Lyon, France

**Keywords:** vaccination, economic evaluation, intangible benefits, healthcare system, antibiotic resistance, complications

## Abstract

Previous economic evaluations of new vaccines largely focussed on a narrow set of benefit categories, including primarily health gains and disease-related medical cost-savings, which probably resulted in underestimates of the true value of these vaccines. Other economic benefits of vaccines could be considered to assess the full economic value of vaccination, such as, for example, impact of the human papillomavirus vaccine on women's fertility through the decrease in precancerous lesions and, therefore, in the number of diagnostic and treatment interventions, which can be associated with an increased risk of subsequent pregnancy complications. Vaccines’ impact on resource allocation at hospital level or on antimicrobial resistance, such as pneumococcal conjugate vaccines that have substantially reduced infections due to antimicrobial non-susceptible strains, thereby rendering the residual disease easier to treat, are other examples of intangible benefits of vaccination. These benefits are generally not considered in economic evaluations because they may not be immediately visible and are difficult to quantify. However, they should be taken into consideration in health technology assessments to enable those responsible for healthcare policies to make well-informed decisions on vaccination.

National policymakers and international organisations commonly use the results of economic evaluation in the frame of their health technology assessments to inform spending decisions on vaccination programmes. However, existing economic evaluations tend to focus on a narrow set of benefits with a narrow perspective, including primarily health gains and disease-related medical cost-savings. They do not usually consider the broader benefits, such as outcome-related productivity gains (i.e., improved economic productivity due to prevention of diseases and associated mental and physical disabilities), impact on healthcare system efficiency, or some positive externalities (i.e., prevention of antibiotic resistance). One of the reasons for not considering some of these benefits is that many of them are ‘invisible’ and/or difficult to quantify. Some economic benefits related to outcome productivity gains have been discussed in other papers in this special issue (1, 2). The objective of this article is to highlight some intangible benefits of vaccination that are usually neglected in traditional economic evaluations, and which could contribute to a more accurate assessment of the full value of vaccination.

## HPV vaccination impact on fertility and neonatal morbidity

The first example of a neglected benefit of vaccination is the impact of human papillomavirus (HPV) vaccination on women's fertility, and neonatal prematurity. Cervical intraepithelial neoplasia (CIN) is the precursor of invasive cervical cancer and is due to HPV infection. Conisation, a standard treatment for high-grade CIN, is associated with an increased risk of subsequent pregnancy complications, such as premature delivery and possible subsequent life-long disability. Although HPV vaccination has the potential to decrease neonatal morbidity and mortality, this has not been taken into account in published cost-effectiveness models (3).

A recent German study estimated that if HPV 16/18 vaccines were to be considered as ‘vaccines against conisation-related neonatal morbidity and mortality’ only (i.e., not considering vaccination's impact of cervical cancer), they would have the potential to be cost-effective (4). This adds to the well-recognised benefits of HPV vaccination on cervical cancer and CIN. Future cost-effectiveness studies should take this significant benefit into account when assessing the economic value of HPV vaccination to ensure that policymakers have accurate and relevant information to reach well-founded decisions regarding HPV vaccination programmes.

## Reduction in disease severity, complications, and comorbidities

Vaccination prevents people from being infected and, thus, developing disease but it can also prevent severe complications. For example, there is increasing evidence supporting the use of influenza vaccination for the secondary prevention of myocardial infarction, which is now usually accounted for in economic evaluations 5,6. However, these benefits are not yet considered for two other vaccines which could also potentially reduce myocardial infarction and stroke risk in those aged ≥50 years:

- *Herpes zoster (HZ) vaccine against shingles*: it has been reported that people who develop shingles are at about a 30% higher risk for stroke (7).
- *Pneumococcal vaccines*: the results from a large hospital-based case–control study suggest that pneumococcal vaccination was associated with a 50% lower risk of myocardial infarction 2 years after vaccination (8).

Recently, the World Health Organization concluded that measles vaccination could be associated with large reductions in all-cause childhood mortality (9). This is supported by results from a recent study analysing pre- and post-vaccination data in Denmark, UK, and United States, providing evidence for a generalised prolonged (2–3 years) impact of measles infection on subsequent mortality from other infectious diseases, thus suggesting that measles vaccination might also produce strong and durable herd protection against all-cause infectious diseases (10). This implies that mortality and morbidity reductions linked to measles vaccination might be much greater than previously considered and reinforce the importance of measles vaccination in a global context.

Lastly, vaccines may also reduce severity of infection and thereby the amount of healthcare resources used. Disease can occur in vaccinated individuals as vaccines are usually not 100% efficacious; however, in such cases, the disease is usually milder than in non-vaccinated individuals (11). For example, in a German efficacy study of an acellular pertussis vaccine, vaccinated individuals who developed whooping cough had a significantly shorter duration of chronic cough than controls (12).

## Reduction of pressure on healthcare systems

Research highlights the importance of hospitals being able to cope in the event of unexpectedly large demands, for example, from an epidemic outbreak of an infectious disease such as swine influenza (13). This results in capacity being held back in preparation for such events, which represents around 5% of the total cost of an emergency admission (14).

In addition to reducing healthcare costs, vaccination can help to strengthen the sustainability of healthcare systems, especially at the hospital level. For example, vaccines such as influenza and rotavirus vaccines can contribute to a reduction in hospital admissions, thereby enabling a better allocation of resources.

In temperate climates, rotavirus gastro-enteritis (RVGE) coincides with other common childhood epidemics, causing more than 40% of the total burden of infant hospitalisations (i.e., respiratory syncytial virus, influenza) occurring over the same seasonal period, leading to the so-called ‘winter chaos’ (15). Coincidence of these epidemics places healthcare systems under pressure, causing an increased risk of nosocomial infections from periodic overcrowding, resulting in additional cases, adverse events, understaffing, lack of beds due to extended hospital stays, and closure of wards to new admissions (16–18). Additional costs resulting from the coincidence of RVGE with other infections are likely to occur in a number of areas that may not be fully captured in cost-effectiveness analyses. Furthermore, the increased burden on hospital capacity can put pressure on staff, affecting their ability to deliver high-quality care to children or delaying planned surgeries for other children (18). Post-vaccination surveillance data have shown a delay in the onset of RVGE epidemics, leading to a reduction in the overall numbers of cases and in the epidemiological overlap, which may decrease workload pressures. For example, in Belgium, following the introduction of rotavirus vaccination, there was a 65–83% reduction in rotavirus hospitalisations and a delay of 4–6 weeks in the onset and peak of the RVGE epidemic (19, 20). Another Belgian study in one paediatric hospital evaluated the potential difference between pre- and post-vaccination periods in hospital pattern and personnel management. The results indicated that bed-day occupancy, bed-day turnover, and unplanned readmissions for acute gastroenteritis were lower in the post-vaccination compared with the pre-vaccination periods, suggesting an improvement in quality of care and a reduced pressure on hospital resources after rotavirus vaccination introduction (21).

The pressure on resources induced by RVGE or influenza for hospitals and emergency departments and the resultant impact on budgets and the ability to deliver care may be underestimated. While disease-related hospitalisation costs are usually included in economic studies, the impact of rotavirus infections on healthcare systems and hospital workload and organisation due to excess nosocomial infections, increased hospital resources, and hospital disruption are not usually considered.

## Contribution of vaccination to antimicrobial resistance

Another example of ‘invisible’ benefits is the role that vaccination can play as part of institution-wide antimicrobial stewardship programmes. Antimicrobials have played an important role in the control of infectious diseases. However, microorganisms are able to mutate, and some of these mutations confer resistance to the antimicrobials. The continuous use of these drugs can lead to the selection of resistant strains, thereby rendering the antimicrobial ineffective. This antimicrobial resistance is a serious threat and leads to increasing healthcare costs, longer hospital stays, treatment failures, and even deaths. In 2009, the ECDC and the EMA estimated that 25,000 Europeans die each year as a direct consequence of a multidrug-resistant infection, with costs estimated at €1.5 billion per year (22). Therefore, any strategy that can reduce the use of antibiotics should be considered as important in a long-term portfolio of measures to combat this global health issue. Both viral and bacterial vaccines have the potential to reduce the community's need for antibiotics. Vaccines reduce the prevalence of infection and, subsequently, the prevalence of disease in the population which, in turn decreases the use of antibiotics. Viral vaccines will reduce the number of viral infections that are erroneously diagnosed as bacterial infections, and thus wrongly treated with antibiotics. Furthermore, many viral infections predispose patients to secondary bacterial infection, which, in the past, often lead to antibiotics being prescribed for the prophylactic prevention of these secondary infections. This is a well-known problem for chickenpox and influenza, for which effective vaccines exist. In the case of chickenpox, one of the most common pathogens for secondary infection is *Staphylococcus aureus* and of particular concern are infections caused by the methicillin-resistant strains (methicillin-resistant *Staphylococcus aureus*), which are responsible for an estimated 150,000 infections every year in the European Union alone (23). Pneumococcal conjugate vaccination (PCV) has been reported to reduce invasive pneumococcal disease caused by antimicrobial non-susceptible vaccine types by 81% in children aged ≤2 years (24). Although in the case of PCV7 vaccine, there was a concomitant increase in disease caused by non-vaccine types, such as the antimicrobial non-susceptible strain, 19A, this did not reverse the overall reduction in disease caused by non-susceptible strains. The inclusion of PCV13 decreased the incidence of serotype 19A, very aggressive and resistant to antimicrobials (25).

A recent review reported that all identified studies showed decreased antibiotic use associated with initiation of vaccination programmes or increased uptake of available vaccines (mainly influenza and pneumococcal vaccines). Reductions in antibiotic use ranged from 5 to 10% in randomised controlled trials, to relative reductions of 64% in epidemiological studies, suggesting that vaccination programmes may reduce antibiotic utilisation and, consequently, antibiotic resistance (26).

In the UK, the Joint Committee on Vaccination and Immunisation noted that whilst new antibiotics and greater stewardship of existing antibiotics in hospitals and primary care were essential, vaccination was equally important in the strategy, by reducing the exposure of the population to potentially unnecessary antibiotic therapy. The committee, therefore, advised that future cost-effectiveness analyses of vaccine programmes should account for the potential benefits associated with reducing antimicrobial use if possible (27).

## Value of vaccination in elderly and polymedicated population

While economic evaluations of vaccines always compare the total costs of a new vaccination programme with the total costs of the current intervention, few evaluations consider the broader benefits of vaccines versus the use of medical treatments such as drugs. Indeed, vaccines not only lead to a reduction in treatment costs by preventing disease, they can also avoid problems associated with long-term treatments and polymedication, particularly in the elderly. For example, patient drug non-adherence rates, especially in the elderly, have been estimated to be between 25 and 75%, and the lack of adherence is estimated to cost European governments about €125 billion per year (28, 29). As discussed in another article in this special issue, the current increase in the population aged ≥60 years leads to the conclusion that, by 2050, the number of older persons (>60) in the world will exceed the number of young people (<15) for the first time in history (2, 30). The elderly often have multiple comorbidities, which, combined with immunosenescence, result in an increased susceptibility to many infectious diseases, and in poorer outcomes after infections which are also strongly associated with unhealthy life style, dietary deficiency, and polymedication (31, 32). Polymedication, which is very frequent in the elderly and patients with chronic conditions, can lead to an increased risk of adverse events or a decrease in treatment efficacy due to potential interactions between prescribed and/or non-prescribed drugs. Systemic or cognitive adverse effects of drugs may also be amplified in older adults, leading to confusion, falls, cardiovascular, or respiratory events. These issues result not only in health consequences to patients but also in high costs to healthcare systems. A recent review suggested that the economic impact of administration errors, inappropriate drug prescription, and poor adherence in elderly patients is substantial, with hospital costs being the main driver (33).

Common infectious diseases in the elderly, such as influenza, pneumococcal infections, or HZ, can be challenging to manage and lead to complications that may contribute to their overall functional decline. As an example, patients with post-herpetic neuralgia (PHN), the most frequent chronic complication of HZ, are often of an advanced age and are likely to have more than one comorbidity, such as cardiovascular disease, diabetes, and chronic obstructive pulmonary disease, for which they receive several medications (34). PHN management frequently requires prolonged combination therapy and various conditions such as renal and hepatic impairment, cardiovascular, cerebrovascular and respiratory disease, and psychiatric conditions may modify the efficacy or the tolerability of prescribed drugs. Therefore, patients should be regularly monitored to assess response to treatment, adverse effects, negative impact on comorbidities, and functional decline (34). Prevention of HZ and PHN with vaccination could offer an effective solution to avoid increasing their risk for adverse events and functional decline.

Although issues, such as non-adherence or potential side effects, may also occur with vaccines’ administration, the limited number of required doses and their good safety profile limit these risks and associated costs. While these issues potentially minimise patients’ benefits through reduced efficacy, potential adverse events, and accelerated functional decline, they represent an additional medical and economic burden that is not usually taken into consideration in economic evaluations of vaccination, in particular in older and polymedicated populations.

## Conclusions

Economic evaluations do not usually take into consideration the lost opportunity for economic growth or savings that can be achieved if broader diseases’ complications and comorbidities are prevented or if resource allocation within the healthcare system is improved. It is believed that if policymakers were to include the appropriate factors for avoiding disease altogether (the intangible benefits of health) in the calculation, the value currently attributed to vaccines would be seen to be underestimated by a factor between 10 and 100 (35).

Broader perspectives may require more extensive data collection. For example, analysis of the economic effects of vaccination on antibiotic resistance will require information that may not be currently available. Evidence on the broader benefits of many health interventions is also very sparse. Understanding the complex relations between health interventions, health outcomes, education, and labour productivity has implications for all types of interventions.

Thus, although some intangible benefits of vaccination may be difficult to quantify, they should be considered in Health Technology Assessments to enable policy makers making well-informed decisions regarding vaccination, taking into account all the benefits for health, healthcare systems, and the wider economy, simultaneously with the cost of vaccination.

## References

[1] Postma M, Carroll S, Brandao A (2015). The societal role of lifelong vaccination. J Market Access Health Policy.

[2] Quilici S, Smith R, Signorelli C (2015). The role of vaccination in economic growth. J Market Access Health Policy.

[3] Huang QT, Zhong M, Gao YF, Huang LP, Huang Q, Wang W (2014). Can HPV vaccine have other health benefits more than cancer prevention? A systematic review of association between cervical HPV infection and preterm birth. J Clin Virol.

[4] Soergel P, Makowski L, Schippert C, Staboulidou I, Hille U, Hillemanns P (2012). The cost efficiency of HPV vaccines is significantly underestimated due to omission of conisation-associated prematurity with neonatal mortality and morbidity. Hum Vaccin Immunother.

[5] Phrommintikul A, Kuanprasert S, Wongcharoen W, Kanjanavanit R, Chaiwarith R, Sukonthasarn A (2011). Influenza vaccination reduces cardiovascular events in patients with acute coronary syndrome. Eur Heart J.

[6] Udell JA, Farkouh ME, Bhatt DL, Cannon CP (2012). Influenza vaccination and reduction of cardiovascular events – A systematic review and meta-analysis (Abstract no: 187). Can J Cardiol.

[7] Kang JH, Ho JD, Chen YH, Lin HC (2009). Increased risk of stroke after a herpes zoster attack: A population-based follow-up study. Stroke.

[8] Lamontagne F, Garant MP, Carvalho JC, Lanthier L, Smieja M, Pilon D (2008). Pneumococcal vaccination and risk of myocardial infarction. CMAJ.

[9] Higgins JPT, Soares-Weiser K, Reingold A Systematic review of the nonspecific effects of BCG, DTP and measles containing vaccines 2014.

[10] Mina MJ, Metcalf CJ, de Swart RL, Osterhaus AD, Grenfell BT (2015). Vaccines. Long-term measles-induced immunomodulation increases overall childhood infectious disease mortality. Science.

[11] Andre FE, Booy R, Bock HL, Clemens J, Datta SK, John TJ (2008). Vaccination greatly reduces disease, disability, death and inequity worldwide. Bull World Health Organ.

[12] Schmitt HJ, von Konig CH, Neiss A, Bogaerts H, Bock HL, Schulte-Wissermann H (1996). Efficacy of acellular pertussis vaccine in early childhood after household exposure. JAMA.

[13] Gaynor M, Anderson GF (1995). Uncertain demand, the structure of hospital costs, and the cost of empty hospital beds. J Health Econ.

[14] Hughes D, McGuire A (2003). Stochastic demand, production responses and hospital costs. J Health Econ.

[15] Bruijning-Verhagen P, Sankatsing V, Kunst A, van den Born C, Bleeker E, Thijsen S (2012). Rotavirus-related hospitalizations are responsible for high seasonal peaks in all-cause pediatric hospitalizations. Pediatr Infect Dis J.

[16] Ogilvie I, Khoury H, Goetghebeur MM, El Khoury AC, Giaquinto C (2012). Burden of community-acquired and nosocomial rotavirus gastroenteritis in the pediatric population of Western Europe: A scoping review. BMC Infect Dis.

[17] Widdowson MA, van Doornum GJ, van der Poel WH, de Boer AS, van de Heide R, Mahdi U (2002). An outbreak of diarrhea in a neonatal medium care unit caused by a novel strain of rotavirus: Investigation using both epidemiologic and microbiological methods. Infect Control Hosp Epidemiol.

[18] Bruggenjurgen B, Lorrot M, Sheppard FR, Remy V (2014). Do current cost-effectiveness analyses reflect the full value of childhood vaccination in Europe?: A rotavirus case study. Hum Vaccin Immunother.

[19] Braeckman T, Van Herck K, Raes M, Vergison A, Sabbe M, Van Damme P (2011). Rotavirus vaccines in Belgium: Policy and impact. Pediatr Infect Dis J.

[20] Patel MM, Steele D, Gentsch JR, Wecker J, Glass RI, Parashar UD (2011). Real-world impact of rotavirus vaccination. Pediatr Infect Dis J.

[21] Standaert B, Alwan A, Strens D, Raes M, Postma MJ (2015). Improvement in hospital quality of care (QoC) after the introduction of rotavirus vaccination: An evaluation study in Belgium. Hum Vaccin Immunother.

[22] EDC/EMEA (2009). The bacterial challenge: Time to react. Contract No.: EMEA/576176/2009.

[23] Kock R, Becker K, Cookson B, van Gemert-Pijnen JE, Harbarth S, Kluytmans J (2010). Methicillin-resistant Staphylococcus aureus (MRSA): Burden of disease and control challenges in Europe. Euro Surveill.

[24] Kyaw MH, Lynfield R, Schaffner W, Craig AS, Hadler J, Reingold A (2006). Effect of introduction of the pneumococcal conjugate vaccine on drug-resistant *Streptococcus pneumoniae*. N Engl J Med.

[25] Picazo J, Ruiz-Contreras J, Casado-Flores J, Giangaspro E, Garcia-de-Miguel MJ, Hernandez-Sampelayo T (2013). Impact of introduction of conjugate vaccines in the vaccination schedule on the incidence of pediatric invasive pneumococcal disease requiring hospitalization in Madrid 2007 to 2011. Pediatr Infect Dis J.

[26] Wilby KJ, Werry D (2012). A review of the effect of immunization programs on antimicrobial utilization. Vaccine.

[27] Joint Committee on Vaccination and Immunisation (2014). Minute of the meeting on 1 October 2014.

[28] EFPIA (2013). Health & wealth: Time for Europe to look forward.

[29] Kusserow RP (1990). Medication regimens: Causes of non-compliance.

[30] Department of Economic and Social Affairs – Population Division (2001). World population ageing 1950–2050 (ST/ESA/SER.A/207).

[31] Aspinall R, Del Giudice G, Effros RB, Grubeck-Loebenstein B, Sambhara S (2007). Challenges for vaccination in the elderly. Immun Ageing.

[32] Ricketson LJ, Nettel-Aguirre A, Vanderkooi OG, Laupland KB, Kellner JD (2013). Factors influencing early and late mortality in adults with invasive pneumococcal disease in Calgary, Canada: A prospective surveillance study. PLoS One.

[33] Chiatti C, Bustacchini S, Furneri G, Mantovani L, Cristiani M, Misuraca C (2012). The economic burden of inappropriate drug prescribing, lack of adherence and compliance, adverse drug events in older people: A systematic review. Drug Saf.

[34] Pickering G, Bertrand I, Marcoux M, Morel V, David L, Macian N (2015). Treatment of postherpetic neuralgia: A touchy mission in elderly patients, particularly in those with chronic diseases and polypharmacy.

[35] Rappuoli R, Miller HI, Falkow S (2002). Medicine. The intangible value of vaccination. Science.

# Sub article: Vaccination: short- to long-term benefits from investment

**DOI:** 10.3402/jmahp.v3.27279

**Published:** 2015-08-12

**Authors:** Stuart Carroll, Amós José García Rojas, Anna H. Glenngård, Carmen Marin

**Affiliations:** 1 Sanofi Pasteur MSD, Maidenhead, UK; 2 Spanish Association for Vaccinology, Spain; 3 Lund University School of Economics and Management, Lund, Sweden; 4 Sanofi Pasteur MSD, Madrid, Spain

**Keywords:** Vaccination, investment, short-term, benefits, costs, budget

## Abstract

In the context of current economic difficulties across Europe, accurate budgeting and resource allocation have become increasingly important. Vaccination programmes can respond to the needs of governments to budget with confidence. It may be more reliable and accurate to forecast budget and resource allocation for a vaccination programme than for unpredictable seasonal disease peaks of infections such as rotavirus gastroenteritis, influenza, and pneumonia. In addition, prevention through vaccination involves low levels of investment relative to the substantial benefits that may be obtained. In France, total lifelong vaccination costs, per fully compliant individual, ranged from €865 to €3,313, covering 12 to 16 diseases, which is comparable to, or lower than, costs of other preventive measures. In addition, effectively implemented vaccination programmes have the potential to generate substantial savings both in the short and in the long term. For example, vaccination programmes for rotavirus, meningitis C, human papillomavirus, influenza, and pneumonia have all been shown to significantly reduce the disease burden, and thus the associated costs, in the first years following vaccination implementation. These programmes demonstrate the potential for health authorities to obtain early, and often substantial, return on investment.

In Europe, a small percentage of national health care budgets is allocated to prevention (3% in average), and only a small part of it is allocated to vaccination. More than three-quarters of OECD countries reported a cut in real-term spending on prevention programmes in 2011 over 2010, and half spent less than in 2008 (1). Indeed, preventive programmes are most vulnerable to budget cuts and restrictions as their benefits may not be always immediately identifiable whilst cuts often focus on short-term financial results. However, any short-term benefits to budgets are likely to be greatly outweighed by the long-term impact on health and spending (1, 2).

Vaccination is often considered a long-term investment only, overlooking the potential short- and medium-term benefits. However, when implemented efficiently, vaccination programmes can also provide early and substantial returns on investment.

In this paper, we will examine the role and advantages of vaccination programmes in health care budget planning, the cost of lifelong vaccination strategies compared with other preventive measures, and the short-term benefits that have been reported for some recently introduced vaccines.

## Vaccination programmes can contribute to accurate health care budgeting

Scarce resources for health make accurate health care budgeting and resource planning essential for achieving important health systems goals. This is essential for achieving value for money and ensuring that budget holders can plan and manage public health expenditures with reasonable precision. It is also fundamental for the sustainability of health care systems that need to respond to the imperatives of continuous reform. Such principles are embedded in European and national guidelines for economic evaluation.

Vaccination programmes can respond to the imperative of helping governments budget with confidence. It is possible for health care systems to accurately forecast the upfront costs of vaccination programmes, or at least to estimate the maximum budget assuming 100% uptake, whereas it is more difficult to precisely forecast and budget for the variable costs of treating vaccine-preventable diseases. Indeed, while disease dynamics and future epidemiology are equally uncertain when modelling drugs and vaccines benefits, predicting treatment costs may be subject to considerable uncertainty since several behavioural and environmental parameters need to be taken into consideration (3).

If we take the example of rotavirus vaccination, from a budget forecast point of view, it is easier to estimate accurately the costs for a vaccination programme than to estimate the variable (and uncertain) costs of treating preventable rotavirus gastroenteritis cases every year, particularly since the incidence and severity of disease vary between seasons. Moreover, these estimated treatment costs may not reflect the full financial burden to health care systems and society as productivity and opportunity costs are very difficult to evaluate. This is also true for other diseases that are associated with annual peaks or seasonality, such as influenza and pneumonia. In conclusion, compared with treatment-focused interventions that have deferred costs distributed over several years according to the disease epidemiology, prevention-focused strategies have budgetable short-term costs which are concentrated in time. This better predictability of expenses achieved through vaccination could bring an additional ‘safety’ value for budget holders.

## Total direct costs of vaccination throughout life are comparable to or lower than those for other preventive measures

Within health care expenditures, prevention spendings represent a small percentage (<3%) of total expenditures in Europe (1). In France, total expenditure on vaccines represented an estimated 0.3% of total health care expenditure in 2013 (4).

A recent study estimated that the lifelong costs per individual vaccinated in full compliance with national recommendations in France ranged from €524 (men without underlying conditions) to €1,379 (women with underlying conditions) in 2014, covering 12 to 16 diseases, respectively, and from €865 to €3,313 when including administration costs (Fig. 1) (5). It was estimated that lifelong vaccine costs were 4 to 10 times lower than costs of statins in the treatment of hypertension; 7 to 14 times lower than the costs for DPP-4 inhibitors in the treatment of type 2 diabetes; and 6 to 13 times lower than costs for antithrombotic medication in the prevention of recurrent stroke. Hence, prevention through vaccination requires low levels of investment relative to the substantial benefits that can be obtained.

**Fig. 1:** Lifelong vaccination costs per fully compliant individual in France (5).

## Short-term value and rapid return on investment

Policy makers are interested in costs and effectiveness in both the short and the long term when deciding which health care technologies they want to invest in. The impact and potential savings in disease treatment costs due to vaccination programmes often occur many years after the implementation of the programme, which makes cost-effectiveness studies of vaccination programmes subject to some uncertainty before real-world data can confirm their predictions. In addition, the discounting of health outcomes imposed by cost-effectiveness guidelines devalues the long-term benefits of prevention programmes such as vaccination, compared with short-term interventions (6).

However, there are examples of vaccination programmes that generate visible short-term savings. One such example is the recent implementation of rotavirus vaccination in several European countries. Rotavirus is highly contagious and resistant to most soaps and disinfectants. Symptoms often include vomiting and fever as well as diarrhoea which, in severe cases, can result in life-threatening dehydration. A UK cost-effectiveness analysis showed that introducing a rotavirus vaccination programme could pay back between 58 and 96% of the outlay costs for vaccination within the first four years of the programme (Fig. 2) (7). Another study in Italy showed that, as early as the second year after programme introduction, the costs of the vaccine would be more than offset by savings from prevented cases of rotavirus gastroenteritis and reduced number of hospitalisations (8). Savings amounted to €34,440 over 5 years, which is equivalent to €4.64 per child from the perspective of the national health service. The estimated economic impact of introducing the vaccine would have been even higher if costs for absenteeism and loss of productivity had been taken into consideration. In countries where rotavirus vaccination programmes have been implemented, a clear clinical benefit has already been reported, confirming predictions from cost-effectiveness models. The most recent example comes from the newly introduced national infant rotavirus vaccination programme in the United Kingdom (implemented in summer 2013). Data on the impact of the immunisation programme in England after its first full year showed a 71% reduction in laboratory-confirmed cases (9). Significant reductions were also seen for the number of GP-reported cases and those from Accident & Emergency departments.

**Fig. 2:** Budget impact analysis showing the cumulative mean annual percentage payback predicted for introduction of rotavirus vaccination in England and Wales (7). (a) Immediate vaccine immunity waning after vaccination and (b) delayed vaccine immunity waning after vaccination. Price for a full-course regimen was assumed to be £60.

Another example of short-term savings from vaccination comes from the experience in the United Kingdom with their meningitis C vaccination programme (Fig. 3) (10). The United Kingdom was the first country to introduce meningococcal serogroup C conjugate vaccination in November 1999. Within 5 and 10 years of vaccine introduction, the number of reported cases declined by 93 and 99%, respectively, compared to baseline. After 10 years, it was estimated that the cumulative savings from the approximately 9,000 cases avoided since the start of the vaccination was £75 million (10, 11). Although this could be an overestimation as the cost per case was calculated based on resources required for treating those aged between 0 and 17 years (who incur higher long-term treatment costs in the event of complications), substantial savings are confirmed.

**Fig. 3:** Annual number of meningitis C cases from 1998/1999 to 2009/2010* (10). *Provisional data.

Quadrivalent human papillomavirus (4HPV) vaccination programmes provide another example of short-term value and return on investment. Prior to introduction, a model applied to Norway estimated that 90% of all costs avoided during the first 5 years of a quadrivalent HPV vaccination programme in 12- to 24-year-old girls would be due to avoided HPV6/11 diseases (i.e., genital warts, or GWs) (12). The findings from this model were also confirmed by real-world data in settings where high coverage rates were achieved. For example, between 2007 and 2009, Australia introduced HPV quadrivalent vaccination for all females aged to 12–26 years. There was an uptake of about 70% for three doses in the school-age cohort (13). By the end of 2009, an Australian nationwide surveillance programme using data from eight sexual health centres reported a 59% reduction in the number of new cases of GWs in females aged 12–26 years (13). By mid-2011, 4 years after the start of the vaccination programme, another Australian study showed that large reductions in new GW episodes occurred in women aged 21 years (92.6%) and 21–30 years (72.6%) compared with the pre-vaccination period (14). This study also reported an important decline in GWs in heterosexual men, which is most likely attributable to an indirect ‘herd protection’ as they were not part of the HPV vaccine target population.

These examples of vaccination short-term benefits confirm that, in addition to long-term impact on population health, vaccination can also provide rapid and substantial returns on investment if implemented efficiently.

## Conclusions

Vaccination plays an important role in public health strategies conferring predictability and confidence in budgeting. Disease prevention through vaccination requires low levels of investment relative to the substantial short-term and long-term benefits that can be obtained. Although vaccines provide a range of long-term benefits, it is important that policy makers consider also the short-term benefits when taking decisions about the introduction of vaccination. There is, therefore, a strong case for consideration of return on investment analyses, which may better reflect the short-term benefits of vaccination, as an addition to cost-effectiveness analyses which focus on a longer time horizon and may devalue the long-term benefits of prevention programmes compared to the short-term benefits of curative programmes. This is essential for resource allocation decisions concerning public health budgets and wider health care interventions.

## References

[1] OECD Health at a Glance 2013: OECD indicators.

[2] O'Riordan M, Fitzpatrick F (2015). The impact of economic recession on infection prevention and control. J Hosp Infect.

[3] Mao L, Yang Y, Qiu Y, Yang Y (2012). Annual economic impacts of seasonal influenza on US counties: Spatial heterogeneity and patterns. Int J Health Geogr.

[4] Zaidman C, Roussel R, Le Garrec MA, Bouvet M, Solard J, Mikou M (2014). Comptes Nationaux de la Santé 2013.

[5] Baron-Papillon F, Cornier M, Remy V, Chriv E (2014). What are the lifelong costs of vaccinating one individual? The French case (Abstrct PIN48). Value Health.

[6] Bonneux L, Birnie E (2001). The discount rate in the economic evaluation of prevention: A thought experiment. J Epidemiol Community Health.

[7] Atkins KE, Shim E, Carroll S, Quilici S, Galvani AP (2012). The cost-effectiveness of pentavalent rotavirus vaccination in England and Wales. Vaccine.

[8] Vitale F, Barbieri M, Dirodi B, Vitali Rosati G, Franco E (2013). [A full economic evaluation of extensive vaccination against rotavirus with RIX4414 vaccine at national and regional level in Italy]. Ann Ig.

[9] Public Health England (2014). Early evidence of the impact of the national rotavirus immunisation programme. HPR.

[10] Public Health England Invasive meningococcal infections laboratory reports in England and Wales.

[11] Public Health England 10 years of meningitis C vaccine: Outstanding health protection measure of the past decade.

[12] Dasbach E, Insinga R, Elbasha E (2008). The epidemiological and economic impact of a quadrivalent human papillomavirus vaccine (6/11/16/18) in the UK. BJOG.

[13] Donovan B, Franklin N, Guy R, Grulich AE, Regan DG, Ali H (2011). Quadrivalent human papillomavirus vaccination and trends in genital warts in Australia: Analysis of national sentinel surveillance data. Lancet Infect Dis.

[14] Ali H, Donovan B, Wand H, Read TR, Regan DG, Grulich AE (2013). Genital warts in young Australians five years into national human papillomavirus vaccination programme: National surveillance data. BMJ.

# Sub article: From population to public institutions: what needs to be changed to benefit from the full value of vaccination

**DOI:** 10.3402/jmahp.v3.26965

**Published:** 2015-08-12

**Authors:** Thomas Szucs, Sibilia Quilici, Marina Panfilo

**Affiliations:** 1 European Center of Pharmaceutical Medicine, Institute of Pharmaceutical Medicine, University of Basel, Basel, Switzerland; 2 Sanofi Pasteur MSD, Lyon, France; 3 Sanofi Pasteur MSD, Roma, Italy

**Keywords:** vaccination, outbreaks, value, vaccine industry, economic evaluation, vaccines complexity

## Abstract

The poor perception of the benefits of vaccines, and their subsequent underuse, can result in substantial economic, societal, and political burden. Adequate support and communication from health authorities and governments is essential to promote the benefits of vaccination and reduce the risk of infectious diseases outbreaks. Cost-containment policies in the vaccine procurement processes could also be a threat to the long-term sustainability of the vaccine industry and manufacturing sites in Europe. Biologicals, such as vaccines, are highly technical and complex products to manufacture and only a few industries are engaged in this activity. Developing incentives to encourage vaccine manufacturers and identifying means of taking into consideration the specificities of vaccines in economic evaluations could allow the full value of vaccination to be appreciated. In conclusion, governments, international agencies, and other stakeholders have an important role to play to help society regain confidence in vaccination and ensure that the benefits of vaccination programmes are fully recognised and valued.

Vaccination has been shown to be a valuable intervention from medical, economic, and societal points of views. It is one of the rare interventions that has had such an important cross-sectorial contribution and population level impact. Despite this, benefits from vaccines are often poorly appreciated by the public and by governments leading to low vaccination coverage rates and reflecting a ‘societal disinvestment’ in vaccination programmes. In addition, the economic value of vaccines is often underestimated compared with that for curative drugs, as seen by the fact that the worldwide expenditure for vaccines represents only about 3% of the global medicines market (2010 figures) (1); in Italy and France, vaccines represented only 1.2 and 1.8% of the total pharmaceutical public expenditure in 2013 (2, 3).

There has been a substantial increase in healthcare expenses due to the rise of chronic diseases in populations. It is estimated that approximately 75% of Europe's healthcare budget is spent on chronic diseases, accounting for €700 billion annually (4). Although pharmaceutical companies have invested heavily in developing new therapies, very few have invested in the less attractive business potential of vaccines (high degree of complexity, low return on investment, etc.). Consequently, in the United States since 1967, the number of vaccine manufacturers has fallen from 37 to 10. Currently, 80% of the vaccine market is held by five manufacturers (5) with about $750 million spent on research and development compared with $26.4 billion for pharmaceuticals (6, 7). The current lack of recognition of the value of vaccination, increasing anti-vaccine lobbies, and price-driven vaccine purchasing processes (which do not acknowledge the specificity, complexity, and necessary investment for the development of vaccines) can have a short-, medium-, and long-term impact on the sustainability of the vaccine industry in Europe, with important health and economic consequences. This article addresses the need for a change in the perception of vaccines and recognition of their value to maximise their contribution to the promotion of healthier European populations.

## Suboptimal vaccine coverage

Over the past few decades, vaccination has led to the control and even elimination of several vaccine-preventable diseases in Europe. However, outbreaks of preventable diseases, such as measles or pertussis, continue to occur even in countries with well-established vaccination programmes. These outbreaks happen when the level of vaccination coverage in a population is not sufficiently high to contain the pathogen (8). First, this can occur when the population stops adhering to vaccination programmes because individuals do not fear a disease they do not know anymore. Thus, vaccines are ‘victims of their own success’ and outbreaks are due to the failure to vaccinate the susceptible population (9). Second, as for any biological product, adverse reactions, although extremely rare (1 per million doses administered), can have negative effects on public trust in vaccination, thus fear of the risk of the vaccine is greater than the fear of having the disease (10). Third, difference in vaccines type (i.e., live attenuated vs. inactivated vaccines), trade-off between efficacy and safety (i.e., whole cell vs. acellular), and the different capacity of individuals to respond efficiently to vaccines (i.e., decreased immunogenicity in the elderly) means that vaccines are not 100% efficacious and their duration of protection varies. This is why there are booster strategies for some vaccines in national vaccination programmes. For example, the increased incidence of pertussis, despite high vaccination coverage in infants, maybe due to several reasons, including increased diagnostic testing due to higher disease awareness and improved laboratory diagnostics and surveillance (11). Furthermore, neither natural infection nor vaccination provides lifelong immunity against pertussis, thus vaccination strategies to vaccinate almost exclusively children may not be sufficient and should be accompanied by booster vaccination in adolescents and adults (11, 12). The limited duration or level of protection that exists for some vaccines reinforces the need to achieve high coverage rates to maximise the direct and indirect effects of vaccination within the population.

Erosion of parents’ trust in vaccines is also linked to the many controversies and scares that have been brought to the public attention by the media and kept alive by anti-vaccine lobbies (13, 14). The pertussis vaccine controversy that started in the mid-1970s in the United Kingdom after the publication of a report alleging that 36 children suffered serious neurological conditions following DTP vaccination, is often considered as the re-activation of anti-vaccination opposition in modern days. This report attracted much media attention and waves of public concerns and, by 1977, vaccination coverage had declined from 77 to 33%. This was followed by three major epidemics of pertussis with over 100,000 cases and the deaths of at least 36 children. Nearly 25 years after the DTP controversy, the United Kingdom faced another major public crisis in vaccine confidence, due to an alleged link between measles-mumps-rubella (MMR) vaccination and autism (15). Measles vaccination rates in children fell from over 90% in 1997 to less than 80% in 2004 (13). Lastly, poorly managed vaccination campaigns in some countries have also led to widespread mistrust of vaccines and government vaccination programmes (16, 17).

In many countries in the WHO European Region where vaccination uptake has decreased over recent years, there has been a rise in the number of measles cases, with over 90,000 cases of measles in the past 3 years (Table 1) (18). In their recent initiative, WHO Europe reaffirmed the economic burden of these preventable diseases (19):

- A measles outbreak in Duisburg, Germany, in 2006 led to 311 schoolchildren missing a total of 2,854 school days, and 30 working adults missing 301 work days. The average cost of a measles case in Germany was estimated to be €520.
- A study in 10 Western European countries revealed that mothers missed between 8 and 24 h of work to care for their children with uncomplicated measles.
- In 2002–2003, the direct cost of measles incurred by the Italian national health service was between €17.6 million and €22 million. This would have paid for vaccinating up to 1.9 million children, which would also have prevented many cases of mumps and rubella. The 5,154 hospitalisations during this period costed about €8.8 million (18).
- In the United Kingdom, reduced MMR vaccination uptake resulted in 2,514 cases of measles being reported in 2008/2009, which was 2,366 more than in 1998/1999 (20). The cost of treating these extra cases was estimated at £587,500 over the 2-year period. These calculations were based on estimated average cost per measles case in industrialised countries and the number of measles cases reported by Public Health England (20, 21).

**Table 1:** Number of reported cases of vaccine-preventable diseases in the European region

	1980	2011	2012	2013
Measles	851,849	37,421	37,073	26,982
Rubella	No data	621,039	9,672	30,509

From Ref. (11).

Suboptimal vaccine coverage can therefore lead to disease outbreaks that can be costly, not only for healthcare resources but also for societal considerations. They affect not only individuals but also the society as a whole. In the current age of information, even a small outbreak can have an impact on the confidence of businesses, families, and the wider society, leading to a decrease in investments, tourism, and consumption (22).

## Suboptimal economic evaluations

Pricing and reimbursement decisions for pharmaceutical products are partly based on cost-effectiveness criteria. Cost-effectiveness analysis is a very useful tool for decision-makers to help prioritising access to health technologies while taking into consideration budget constraints. Cost-effectiveness is generally considered from the healthcare provider's perspective, so as to optimise national healthcare budgets. This methodology has been widely used for vaccines assessment but is now considered to be too narrow, given that vaccines benefits are population-wide, cross-sectorial, and go beyond their direct impact on healthcare systems. Results from research are increasingly providing evidence on the wider value of vaccination as discussed in two other articles in this special issue (23–27).

Although there is consensus that vaccines are vastly cost-effective, it does not seem sufficient to attract more public and private investments. However, the whole economy of a country can be impacted in the event of an outbreak of serious infectious diseases, such as pandemic influenza. It has been estimated that the largest impact of a pandemic influenza in the United Kingdom would be in the sectors of meat and livestock, processed foods, textiles, manufacturing, transport and communications, with a loss of up to 2.5% of the gross domestic product (GDP) (Fig. 1) (28). In addition to the preventable disease and healthcare costs, the preventable GDP loss needs to be taken into consideration. Potential means have been identified to increase incentives to invest in vaccines and to account more accurately for the full economic value of vaccines. In particular, Health Authorities could use a long-term horizon and a lower discount rate for vaccines than for drugs when evaluating health technologies or assign a higher cost-effectiveness threshold to take into consideration some of their intangible value (1). Indeed, it has been estimated that if ‘intangible’ values were to be included in a cost–benefit analysis, the cost–benefit ratio would be improved by a factor from 10 to 100, thus providing a rationale to invest in vaccine development (29). Not taking the full economic return from vaccines into consideration can therefore result in an underestimation of their cost-effectiveness and may delay the access of citizens to effective preventive measures.

**Fig. 1:** Impact of pandemic influenza on different sectors of UK gross domestic product (GDP) (28).

## Suboptimal procurement processes

Vaccines are highly technical and complicated products requiring a lengthy and expensive manufacturing process with specialised handling, quality control, and procurement procedures. Their biological nature means that each production cycle corresponds to the manufacturing of a new vaccine which leads to major challenges in terms of reproducibility and consistency and to a high level of uncertainty at any time during the process. Consequently, only few players have developed the expertise to deliver to a market with a steadily increasing demand. Cost-containment efforts in European countries and procurement through tenders awarded based solely on lowest price can also threaten the short- and long-term ability of the European vaccines industry to meet the vaccine needs of countries in the European Union. Shortages of vaccines have occurred in the past and still continue to occur today. The reasons for these shortages are multi-factorial, including some companies leaving the vaccine market place because they consider it is no longer profitable, manufacturing issues or difficulties in stockpiling due to expiry date, and cold chain management. These shortages can affect millions of at-risk individuals, such as infants, and can lead to delay of certain vaccinations, leading to an increased risk for vaccine-preventable disease. The costs associated with shortages are thought to be high. Using a model developed by the CDC and applied to pertussis in the United States in 2007, in the absence of stockpile, shortage could lead to 53 pertussis hospitalisations in the best case scenario (i.e., 10% shortage and low incidence of pertussis) and up to 4,183 hospitalisations in the worst case scenario (i.e., 40% shortage and high incidence of pertussis) (30). The cost of hospitalisation for pertussis was estimated to be $6,577 in 2007 for an infant (31). This resulted in an estimated cost ranging from $350,000 to $27 million, depending on the level of dose shortage, that is, 10–40%, due to insufficient stockpiles (30).

The 2011–2012 influenza season was marked by a substantial shortage of seasonal influenza vaccine in several European regions due to anomalies identified in certain vaccine batches. The shortages most notably affected countries where procurement, either nationally or in specific regions, was sourced from a single supplier, such as in Spain, Germany, and Italy (32, 33).

These procurement practices can therefore increase the risk of vaccine shortages by discouraging market entry into countries where the price does not provide long-term industry incentives in quality and innovation, and by limiting the options to approach other suppliers should those who are contracted experience production difficulties.

## Low prices can result in inefficient long-term economic dynamics

Current public procurement systems for vaccines are essentially managed by budget holders whose objective is to obtain the best quality product at the lowest possible price to afford the quantities necessary to cover large populations. They usually do not consider the macro-economic income of the vaccine industry within the global economy. In the longer term, low prices can lead to perverse economic dynamics as they do not provide adequate resources to be invested in improving vaccine technology and expanding disease prevention through research and development. For example, over the past 3 years, the average selling price of trivalent influenza vaccines sold in bulk in Europe has fallen by half to the current average selling price of around €3.50 per dose (34). Cost-containment and tender processes, based solely on the criteria of the lowest price, are detrimental to manufacturers who, in the long run, will not obtain the necessary return on investment to remain in the market and may reduce investment in research, thus leading to reduced innovation in this sector.

The situation of under-priced vaccines in Western countries has previously resulted in a critical procurement situation with unprecedented impact for vaccine supply in developing countries. Between 1998 and 2001, 70% of UNICEF suppliers stopped part or all of their paediatric vaccine production due to lack of business profitability. With only two manufacturers left on the market, vaccination programmes were placed at significant risk in terms of supply capacity but also in terms of price stability in the absence of adequate competition. It is only recently that manufacturers have returned following the implementation of a procurement strategy by GAVI spanning several years which thus results in a more sustainable marketplace in developing countries (35).

This illustrates that the differences between vaccines and drugs in terms of complexity and production costs are not fully understood. In comparison with drugs, vaccines are highly complex products produced in extensively regulated facilities. Research on production technology is needed to bring down production costs. The same is true for development of new vaccines, new combination vaccines, and new delivery systems. These funds are not available when prices do not support resources for research and development (36). As a result, there will be increasingly fewer players on the market place, which will impact the public health and the socio-economic fabric of the country.

## Conclusion

The combined effect of mistrust of the population in vaccination, the suboptimal economic evaluation methods that only account for the healthcare-related benefits of vaccines, and the lack of recognition by governments and other purchasers of the complexity of vaccine manufacturing process can result in no wealth creation. This is exactly the opposite of what is expected from preventive therapies such as vaccines that are developed to maintain population health, and health is wealth. Indeed, if payers do not see all the benefits from vaccination, but only the costs, they will underinvest. If doctors do not see diseases prevented by vaccination, but only the time spent in monitoring vaccination programmes, they will not understand the importance in their practice. If parents no longer see children sick with serious infectious diseases, but hear only about the ‘dangers’ of vaccination, they will be less willing to have their healthy children vaccinated. Therefore, adequate support and communication from health authorities and governments is necessary to promote the benefits of vaccination and reduce the risk of outbreaks of infectious diseases that are a threat for national public health and economy.

More incentives for the development of vaccines could also contribute to building a healthier society, instead of a society of chronically sick people, by assigning more economic value to disease prevention (29). A number of measures to make vaccines more attractive to industry have been identified. For example, vaccine development could benefit from tax credits and public–private partnerships, and vaccines’ specificities could be taken into consideration in economic evaluations. While cost-effectiveness analyses are useful to inform decision-makers about the efficiency of vaccines, they should also be coupled with other economic evaluations accounting for the broader impact of vaccination on the society and the economy as a whole and estimating the return on investment of vaccination programmes to inform about their affordability.

In conclusion, governments, international agencies, and other stakeholders, such as the medical community, have an important role to play to help society regain confidence in vaccination and ensure that the full benefits of vaccination programmes are fully recognised and valued.

## References

[1] Regnier SA, Huels J (2013). Drug versus vaccine investment: A modelled comparison of economic incentives. Cost Eff Resour Alloc.

[2] Zaidman C, Roussel R, Le Garrec MA, Bouvet M, Solard J, Mikou M (2014). National health accounts in 2013.

[3] Italian Medicines Agency (2013). Drug use in Italy. National report 2013.

[4] EFPIA Health & wealth: Time for Europe to look forward 2013.

[5] Sahoo A (2011). Vaccines 2011: Market analysis, key players and critical trends in a fast-changing industry.

[6] Berman S, Giffin RB (2004). Global perspectives on vaccine financing. Expert Rev Vaccines.

[7] Whitehead P, Pasternak A (2002). Lessons learned: New procurement strategies for vaccines. Final Report to the GAVI Board.

[8] World Health Organisation Health topics: Disease outbreaks 2015.

[9] Centers for Disease Control and Prevention (2011). Increased transmission and outbreaks of measles – European Region, 2011. MMWR.

[10] Bloom DE, Canning D, Weston M (2005). The value of vaccination. World Econ.

[11] Wicker S, Maltezou HC (2014). Vaccine-preventable diseases in Europe: Where do we stand?. Expert Rev Vaccines.

[12] Plotkin SA (2014). The pertussis problem. Clin Infect Dis.

[13] Dube E, Vivion M, MacDonald NE (2015). Vaccine hesitancy, vaccine refusal and the anti-vaccine movement: Influence, impact and implications. Expert Rev Vaccines.

[14] Gangarosa EJ, Galazka AM, Wolfe CR, Phillips LM, Gangarosa RE, Miller E (1998). Impact of anti-vaccine movements on pertussis control: The untold story. Lancet.

[15] Flaherty DK (2011). The vaccine-autism connection: A public health crisis caused by unethical medical practices and fraudulent science. Ann Pharmacother.

[16] ECDC Individual decision-making and childhood vaccination.

[17] UNICEF (2013). Tracking anti-vaccination sentiment in eastern European social media networks.

[18] World Health Organisation (2014). WHO vaccine-preventable disease monitoring system, 2014 global summary.

[19] World Health Organisation Measles costs 2013.

[20] Public Health England Measles notifications (confirmed cases) England and Wales 1995 – 2013 by quarter 2014.

[21] Carabin H, Edmunds WJ, Gyldmark M, Beutels P, Levy-Bruhl D, Salo H (2003). The cost of measles in industrialised countries. Vaccine.

[22] Bell C, Lewis M (2005). The economic implications of epidemics old and new. World Econ.

[23] Bonanni P, Picazo J, Remy V (2015). The intangible benefits of vaccination – what is the true economic value of vaccination?. J Market Access Health Policy.

[24] Quilici S, Smith R, Signorelli C (2015). The role of vaccination in economic growth. JMAPH.

[25] Barnighausen T, Bloom DE, Cafiero ET, O'Brien JC (2012). Economic evaluation of vaccination: Capturing the full benefits, with an application to human papillomavirus. Clin Microbiol Infect.

[26] Barnighausen T, Bloom DE, Cafiero-Fonseca ET, O'Brien JC (2014). Valuing vaccination. Proc Natl Acad Sci USA.

[27] Kotsopoulos N, Connolly MP (2014). Is the gap between micro- and macroeconomic assessments in health care well understood? The case of vaccination and potential remedies. J Market Access Health Policy.

[28] Smith RD, Keogh-Brown MR, Barnett T, Tait J (2009). The economy-wide impact of pandemic influenza on the UK: A computable general equilibrium modelling experiment. BMJ.

[29] Masignani V, Lattanzi M, Rappuoli R (2003). The value of vaccines. Vaccine.

[30] Shrestha SS, Wallace GS, Meltzer MI (2010). Modeling the national pediatric vaccine stockpile: Supply shortages, health impacts and cost consequences. Vaccine.

[31] Lee GM, Murphy TV, Lett S, Cortese MM, Kretsinger K, Schauer S (2007). Cost effectiveness of pertussis vaccination in adults. Am J Prev Med.

[32] Deutsche Welle Germany moves to cut off vaccine shortage 2012.

[33] Corriere della serra Milano (2012). Milan, batch of influenza vaccine recalled. Factory suspected.

[34] Hirschler B (2013). New flu vaccines offer extra protection - and more profits.

[35] Lorot P (2012). Vaccines, an advantage at the service of France.

[36] Ehreth J (2005). The economics of vaccination from a global perspective: Present and future. 2–3 December, 2004, Vaccines: All things considered, San Francisco, CA, USA. Expert Rev Vaccines.
